# Continual Learning for Histopathology Image Classification in Class-Incremental Learning

**DOI:** 10.3390/diagnostics16111711

**Published:** 2026-06-02

**Authors:** Yuanyuan Wu, Yu Zhao, Anca Ralescu

**Affiliations:** Department of Computer Science, University of Cincinnati, 2901 Woodside Drive, Cincinnati, OH 45221, USA; wu3yy@mail.uc.edu (Y.W.); zhao3y3@ucmail.uc.edu (Y.Z.)

**Keywords:** continual learning, catastrophic forgetting, class-incremental learning, prompt-based learning, digital pathology, histopathology

## Abstract

**Background**: Continual learning (CL) is increasingly important for developing adaptive clinical AI models; however, its application to histopathology remains challenging due to privacy constraints, expanding diagnostic categories, and staining variability. We investigate CL for histopathology image classification under a class-incremental learning (CIL) scenario, where new diagnostic categories are introduced sequentially. **Methods**: We benchmark representative regularization-, replay-, architecture-, and prompt-based CL methods on the NCT-CRC-HE-100K dataset, with additional validation on CRC-HE-7K. We compare four normalization strategies and analyze the effects of replay buffer size and training epochs. In addition to average accuracy and forgetting, we conduct clinical relevance and error analysis using confusion matrices, ROC curves, and misclassification cases, then assess training dynamics and computational efficiency. **Results**: Dataset-level normalization consistently achieves the best performance among the evaluated normalization strategies. Among replay-based methods, DER++ achieves strong performance when previous-task images can be stored and replayed, reaching an average accuracy of 94.77±1.82 and forgetting of 3.66±1.73 with a buffer size of 500 and 50 training epochs. However, it requires higher memory usage, longer training time, and storage of previous samples. Among prompt-based methods, DualPrompt performs best with 5 epochs, reaching an average accuracy of 88.97±0.60 and forgetting of 7.70±1.21 while showing smoother training behavior and lower computational cost. **Conclusions**: Replay-based methods achieve higher accuracy and lower forgetting when exemplar storage and sufficient computational resources are available, but introduce higher computational and privacy costs. Prompt-based methods provide a competitive exemplar-free alternative under privacy- and resource-constrained settings. Dataset-level normalization is also important for stable CL performance in histopathology CIL.

## 1. Introduction

Deep learning has achieved remarkable success in healthcare, particularly in medical image analysis for tasks such as cancer diagnosis from histopathology [1,2], disease detection from radiology images [3], and various classification and segmentation tasks across X-ray [4], CT [3], MRI [5], and ultrasound [6]. Despite these advances, clinical AI models have largely adopted a static “train once, deploy forever” paradigm in which models cannot adapt continuously to expanding diagnostic categories, evolving data distributions caused by new acquisition devices, or gradual distribution shifts that arise as new patient data are collected over time. In dynamic real-world healthcare scenarios, clinical AI models increasingly require the ability to continually integrate new medical information while preserving previously acquired knowledge.

Histopathology image examination is widely recognized as the gold standard for making final diagnoses of various human lesions [7]. These images are obtained from microscopic views of tissue sections stained with hematoxylin and eosin (H&E), and a single slide may contain several hundred thousand individual cells [8]. However, applying CL to histopathology presents unique challenges: first, annotated tissue categories are frequently expanded over time as new clinical knowledge is established, requiring models to incorporate novel classes without forgetting previously learned ones; second, histopathology images show substantial staining and appearance variability across patients and acquisition sites, which intensifies CF under incremental updates [9]; third, strict privacy regulations often restrict long-term storage of patient data, limiting the feasibility of replay-based CL methods in medical environments.

Together, these challenges define the practical context in which CL systems must operate for histopathology images. In this work, we explicitly focus on the CIL setting, where the label space evolves over time and new diagnostic categories are introduced. While staining and site variability represent important sources of distribution shift that further complicate CL in practice, explicitly modeling such shifts corresponds to a different CL setting that focuses on changes in the input distribution rather than the label space, which falls outside the scope of this study. Instead, we study CIL under realistic conditions that naturally include appearance variability without explicitly modeling domain-level shifts, allowing us to isolate and systematically benchmark the behavior of different CL paradigms under evolving label spaces. Accordingly, we adopt NCT-CRC-HE-100K as our benchmark. This large (100,000 images), diverse (nine classes), and well-curated dataset provides diverse tissue types, realistic staining variability, and wide usage in computational pathology research [10].

Conventional CL approaches are commonly grouped into regularization-based, replay-based, architecture-based, and prompt-based categories [11,12]. The first three CL types represent traditional methods, whereas the last type reflects more recent developments in CL. Regularization-based methods, including LwF [13], Online EWC [14], and SI [11], constrain parameter updates to preserve previous knowledge. Replay-based methods such as ER [15], DER, and DER++ [16] alleviate CF by revisiting stored past samples. Architecture-based methods allocate task-specific model parameters or dynamically expand the model to prevent interference across tasks; in this work, we include DyTox [17] as a representative architecture-based method, as it supports CIL without requiring task identities at inference and offers a more scalable design compared to earlier expansion-based approaches. Traditional CL approaches also often train models from scratch as new tasks arrive, making them highly susceptible to CF. With the rapid progress in large-scale representation learning, pretrained models have recently gained significant attention in CL [18,19]. Leveraging pretrained representations can reduce dependence on replay buffers or strict regularization, and can often enhance both stability on past tasks and plasticity when learning new ones.

Given these benefits of pretrained representations, prompt-based CL methods naturally build upon this paradigm by keeping the backbone frozen and learning only a small set of prompts. Representative examples include L2P [20], DualPrompt [21], and CODA-Prompt [22], all of which avoid the need for task identities or stored exemplars at inference time. Despite their avoidance of memory buffers, prompt-based methods have demonstrated competitive performance compared to replay-based techniques [20,21,22]. These properties make prompt-based CL especially suitable for exemplar-free CL in medical scenarios [12], particularly in digital pathology deployments where privacy constraints prohibit retaining patient data [23] and where new diagnostic categories emerge over time.

We evaluate DER++ and DualPrompt using four normalization strategies: ImageNet normalization, dataset-level normalization [24], per-image normalization [25], and Macenko stain normalization [26]. Because histopathology images differ substantially from natural images in terms of color and distribution, ImageNet normalization is often suboptimal for H&E data. Our results show that DER++ and DualPrompt perform best with dataset-level normalization. Therefore, we adopt this normalization strategy for all subsequent experiments due to its consistently superior performance across CL methods. Moreover, our experimental results show that larger buffer sizes and longer training epochs enable replay-based methods (ER, DER, and DER++) to achieve the highest overall performance. For example, DER++ attains an average accuracy of 94.77±1.82 and a forgetting of 3.66±1.73 when trained for 50 epochs with a buffer size of 500. In contrast, prompt-based methods such as L2P and DualPrompt attain the highest training efficiency with only a few epochs, as they require updating only a small set of prompt parameters rather than the full model. Although CODA-Prompt is a prompt-based approach, it requires more training epochs to reach competitive performance. Among prompt-based methods, DualPrompt performs the best in our experiments, achieving an average accuracy of 88.97±0.60 and a forgetting of 7.70±1.21 at five training epochs.

Our work provides a comprehensive benchmark of prompt-based CL methods alongside representative traditional CL approaches for histopathology image classification under the CIL scenario. The key contributions are summarized as follows:

- We benchmark representative regularization-based, replay-based, architecture-based, and prompt-based CL methods under a realistic CIL setting on the NCT-CRC-HE-100K and CRC-HE-7K histopathology datasets.
- We compare four commonly used normalization strategies and show that dataset-level normalization consistently performs best, highlighting its importance for CL in histopathology images.
- We analyze the practical behavior of CL methods under realistic computational and clinical constraints, covering replay buffer size, training epochs, clinical relevance and error analysis, training dynamics, and training time.

## 2. Related Work

CL aims to allow models to acquire knowledge from a sequence of new tasks without suffering from CF. CF refers to degradation in performance on previously learned tasks when a model is updated with new ones [27]. Achieving an effective balance between stability and plasticity is a main challenge in CL [28,29]. Stability helps to preserve prior knowledge and mitigate forgetting, while plasticity ensures that the model can effectively learn new tasks. Among various CL scenarios [30] in the computer vision field, the most challenging and realistic one is the CIL setting. In this setting, a model must continually learn new classes without knowing its task identity during inference time. Importantly, CIL has strong clinical relevance in medical imaging, where new disease categories or tissue types are progressively introduced in real workflows; this requires continuous model updates while maintaining diagnostic performance on prior categories [31,32]. Motivated by this practical scenario, our study focuses on CIL for histopathology image classification.

In recent years, several surveys have provided overviews of CL from the perspectives of both theoretical foundations and practical application. Qu et al. [11] presented a taxonomy-oriented survey of CL theory, focusing on fundamental challenges such as CF and stability–plasticity tradeoffs. Although their review thoroughly discussed evaluation protocols and methodological frameworks, it did not delve into domain-specific applications. Wang et al. [33] further broadened the scope by covering a wide range of CL techniques across multiple domains, including computer vision, reinforcement learning, and natural language processing. Although highly comprehensive in scope, this review remains theoretical and domain-general, lacking discussion of clinical deployment and regulatory considerations. Kumari et al. [12] provided a comprehensive overview of CL methods for medical imaging analysis, emphasizing the need for CL in medical settings where models must adapt to evolving data distributions and new tasks without suffering from CF. While these surveys outline the development of CL methods and highlight the importance of medical applications, they do not include empirical evaluations on real medical datasets.

Wu et al. [34] examined core CL approaches and their suitability for CIL. Their survey used medical imaging examples to illustrate each learning setting and discussed domain-specific challenges in detail, along with commonly used benchmark datasets. They conducted an evaluation of CL methods on MedMNIST [35] and reported their comparative performance. The findings highlighted the advantages of exemplar replay and the emerging potential of prompt-based models as exemplar-free alternatives in privacy-sensitive medical environments. However, the focus was on low-resolution (28×28) datasets, and the authors did not address unique challenges of histopathology images such as staining variability and high-resolution (224×224) tissue structures. In general, prior benchmarks have not investigated how normalization strategies influence CL performance or provided detailed analyses of training time and memory constraints across CL method families. In contrast, our work conducts a comprehensive empirical evaluation on the large-scale NCT-CRC-HE-100K dataset, examines four normalization strategies, and offers method-specific insights into both replay-based and prompt-based CL approaches under realistic clinical constraints.

Lenga et al. [36] used two chest X-ray datasets to investigate two regularization-based CL methods, EWC [27] and LwF [13], in a domain-incremental learning (DIL) scenario. Their experimental results showed that joint learning achieves the highest performance, as expected, while LwF provides competitive results due to the relatively small domain shift across datasets. However, the CL methods evaluated in their study represent early and relatively simple baselines, and as such do not reflect the capabilities of the more advanced state-of-the-art CL approaches considered in our work. Moreover, their experiments focused on DIL, where class labels remain fixed across domains, whereas we study the more challenging CIL setting in which new classes must be learned over time without forgetting previous ones. In addition, our work conducts a deeper empirical analysis relevant to clinical deployment: we compare four normalization strategies in order to determine how image preprocessing affects CL performance, and examine how practical resource constraints such as limited buffer sizes and training time budgets impact different families of CL methods. These analyses provide insight into which CL approaches are most suitable for real-world clinical AI environments.

## 3. Methods

### 3.1. Prompt-Based Methods

In this section, we introduce a family of prompt-based CL methods, including L2P, DualPrompt, and CODA-Prompt. These approaches are motivated by the success of prompt learning in natural language processing (NLP) [37]. In vision-based CL, prompt learning introduces a small set of learnable tokens to guide a pretrained model toward task-relevant representations. During task adaptation, the backbone network is typically kept frozen and used as a generic feature extractor [21], while only the prompt parameters and classifier are updated. These designs provide a lightweight adaptation mechanism that reduces the number of trainable parameters, helping to mitigate CF without full fine-tuning of the backbone.

**Figure 1:**
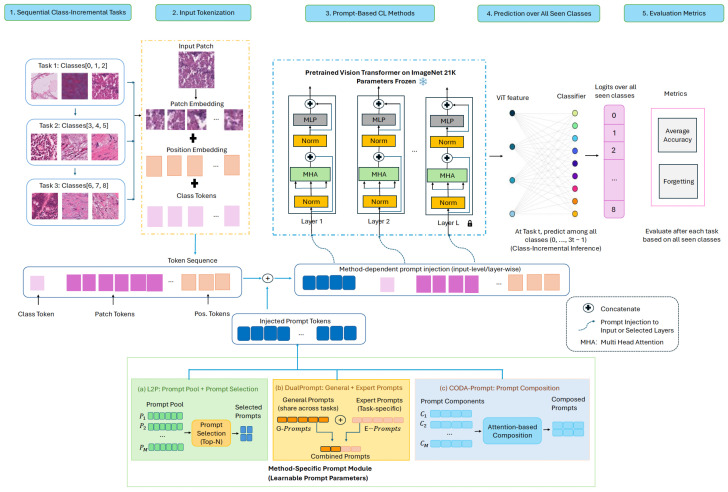
Architectural diagram of prompt-based CL methods for class-incremental histopathology image classification.

Figure 1 summarizes the common workflow of the prompt-based CL methods introduced in this section. Under the CIL setting, new histopathology classes are introduced sequentially across tasks. Each input image patch is first converted into a ViT-compatible token sequence using patch embeddings, positional embeddings, and a class token. A method-specific prompt module then generates task-adaptive prompt tokens, where L2P selects prompts from a learnable prompt pool, DualPrompt combines general and expert prompts, and CODA-Prompt composes prompts from decomposed prompt components. Depending on the method, the generated prompts are prepended to the input token sequence or injected into selected transformer layers, while the pretrained ViT backbone remains frozen. The resulting ViT feature is passed to a classifier that performs prediction over all classes observed so far, and performance is evaluated after each task using average accuracy and forgetting. In this workflow, MHA denotes multi-head attention. Overall, this design reduces the number of trainable parameters and mitigates CF by adapting to new tasks through lightweight prompt parameters rather than full backbone fine-tuning.

Learning to Prompt (L2P) is a prompt-based CL method that introduces a pool of learnable prompts as external memory while keeping the pretrained ViT backbone frozen [20]. For each input image, L2P uses the pretrained ViT model on ImageNet21K to obtain an embedding feature representation $x_{e}$. Next, a query–key matching mechanism is employed to dynamically select prompts. Specifically, each prompt $P_{i}$ is related to a learnable key $k_{i}\in R^{D_{k}}$, forming a prompt pool ${\left(K_{i},P_{i}\right)}_{i=1}^{M}$. The query feature q(x) is extracted from the class token output after feeding the input images to the backbone. Then, the similarity scores between the query and keys are computed using the cosine distance function to select the top-N keys, $s_{i}=\cos\left(q\left(x\right),k_{i}\right)$. Therefore, the selected prompts are added before the input tokens to form the prompted input sequence $x_{p}=\left[P_{s_{1}},...,P_{s_{N}}\right]$ for 1≤N≤M, which is passed through the model and classifier.

L2P optimizes its prompts, keys, and classifier through a combined objective consisting of a supervised classification loss and a key–query alignment loss:

(1) $$L=L\left(g_{\phi }\left(f_{r}^{\mathrm{avg}}\left(x_{p}\right)\right),y\right)+\lambda \sum_{K_{\infty }}\gamma \left(q\left(x\right),k_{s_{i}}\right).$$

In Equation (1), $k_{s_{i}}$ refers to the selected prompt keys, while γ is a similarity-based surrogate loss encouraging keys to be updated closely to query features; thus, the keys are chosen when encountering similar images. Finally, λ is a weighting factor used to balance the classification loss and key learning loss. L2P decouples the prompt learning and query strategies, allowing the model to effectively learn both shared and task-specific prompts. Furthermore, L2P expands task-specific capacity and significantly mitigates CF, achieving a strong stability–plasticity balance without replay.

Dual Prompting for Rehearsal-Free Continual Learning (DualPrompt) extends L2P by introducing two complementary types of prompts to address both generic and task-specific knowledge in CL [21]. This method also keeps the pretrained ViT backbone frozen and uses a prompt pool containing the general prompts that capture transferable knowledge shared across tasks and expert prompts that specialize in task-specific information. Prompts are injected at carefully selected MSA layers via a configurable prompting function.

In more detail, a G-prompt $g\in R^{L_{g}\times D}$ is globally shared across all tasks throughout the CL process and attached to selected transformer layers to encode task-invariant representations. In contrast, an E-prompt $E={e_{t}}_{t=1}^{T}$, where $e_{t}\in R^{L_{e}\times D}$, is associated with each Task-*t*; this specializes the model toward task-unique characteristics. The E-prompt is paired with a learnable key, enabling the most relevant expert prompt via cosine-similarity matching during inference when task identity is unknown. This retrieval mechanism is conceptually inspired by the query–key strategy in L2P, but differs in that keys represent task-level semantics rather than individual prompts.

After learning prompts, it is crucial to determine where and how to insert them into the MSA layers, since different transformer layers capture different types of knowledge [38]. Therefore, the placement and prompting function directly influence how effectively the prompts interact with the backbone representations. G-Prompts and E-Prompts are attached to separate non-overlapping depth ranges to better align with shared versus task-dependent knowledge. In addition, DualPrompt studies two prompting functions: one uses a similar approach as L2P to prepend prompts to Q,K,V inputs from the MSA layer (prompt tuning), while the second divides prompts into $p_{K},p_{V}$ and prepends only to K,V.

To achieve a balance between stability and plasticity, DualPrompt learns the prompts and classifier head through a combined objective. The overall loss consists of a supervised classification term and a constraint term that encourages effective prompt utilization:

(2) $$L=L\left(f_{\phi }\left(f_{g},e_{t}\left(x\right)\right),y\right)+\lambda L_{\mathrm{match}}\left(x,k_{t}\right),\;x\in D_{t}.$$

The first term in Equation (2) is the cross-entropy loss, while the second term $L_{\mathrm{match}}$ makes query features closer to the correct task key for future retrieval. Here, $k_{t}$ is the task key, *g* denotes the G-Prompt parameters, $e_{t}$ is the E-Prompt for Task-t, ϕ is the classification head, and λ controls the balance between the two loss terms.

By decoupling shared and specialized knowledge pathways, DualPrompt improves learned prompt allocation and enhances cross-task generalization compared to L2P. The dual-prompt structure supports robust CL without replay, offering a stronger tradeoff between stability and plasticity under CIL.

Continual Decomposed Attention-based Prompting (CODA-Prompt) [22] is an exemplar-free CL method, similar to L2P and DualPrompt, and is likewise categorized as a prompt-based CL approach. A key distinction between CODA-Prompt and L2P is that all learnable parameters in CODA-Prompt, including prompt components, keys, and attention vectors, are optimized jointly using the standard classification loss rather than splitting them into two separate loss optimizations. This design enables more effective end-to-end optimization and contributes to improved performance.

Instead of selecting a single prompt from a pool, CODA-Prompt introduces a set of learnable prompt components and composes the final prompt as a weighted summation of these components:

(3) $$P=\sum_{m}\alpha_{m}P_{m}.$$

In Equation (3), $P\in R^{L_{p}\times D\times M}$ denotes the collection of prompt components, $P_{m}$ is the *m*-th component, and *M* denotes the number of components. The weighting vector α is computed for each input according to the similarity between the input query and component keys, optionally modulated by attention vectors, enabling CODA-Prompt to construct input-adaptive prompts that encode task-relevant knowledge.

To refine its prompt selection process, CODA-Prompt applies a learnable attention vector to the query embedding. This element-wise interaction highlights features relevant to each prompt component. Then, the similarity between this new type of query and key vectors determines the mixing weights $\alpha_{m}$:

(4) $$\alpha =\gamma \left(q\left(x\right)⊙A,K\right)=\gamma \left(q\left(x\right)⊙A_{1},K_{1}\right),...,\gamma \left(q\left(x\right)⊙A_{M},K_{M}\right).$$

To reduce interference across tasks, CODA-Prompt adds orthogonal constraints on any matrix of prompts, keys, and attention vectors. The loss function is

(5) $$L=L\left(f_{\phi }\left(f_{\theta ,P,K,A}\left(x\right)\right),y\right)+\lambda \left(L_{\mathrm{ortho}}\left(P\right)+L_{\mathrm{ortho}}\left(K\right)+L_{\mathrm{ortho}}\left(A\right)\right),$$

where the first term is the cross-entropy loss; the remaining terms are orthogonality regularizers that encourage prompt-related parameters to span distinct subspaces, which reduces the representational overlap across tasks. Finally, θ denotes the frozen parameters of the pretrained backbone and ϕ represents the learnable parameters of the linear classification head for the current task.

### 3.2. Other Compared Methods

CL has been widely explored in medical image analysis using various paradigms, including regularization-based approaches such as SI as well as replay-based approaches such as ER, DER, and DER++ [39]. In addition, architecture-based methods such as DyTox represent a recent direction in CL. However, despite promising results in the general vision domain, DyTox has not yet been evaluated in medical imaging. To provide a more comprehensive and fair comparison with prompt-based approaches, we introduce DyTox into the medical image CL setting here for the first time.

Online EWC [14] is a scalable modification of EWC [27] that is designed to regularize deep learning model parameters without increasing memory overhead; therefore, it is a regularization-based CL method [12]. Unlike standard EWC, which calculates a separate Fisher information matrix for each task, Online EWC maintains a single consolidated Fisher matrix that is updated after each task; moreover, it uses a decay factor γ, allowing the contribution of older tasks to gradually diminish when new tasks are introduced. This provides a balance between retaining the stability of important knowledge from previous tasks and maintaining plasticity for learning new ones [40], while also ensuring that memory usage remains constant regardless of the number of tasks.

LwF [13] is a CL method [11] based on knowledge distillation. The model is regularized by encouraging predictions on earlier tasks to remain similar to soft targets generated by the previous model while simultaneously optimizing a new task loss. Meanwhile, the distillation loss function helps to preserve learned knowledge through shared feature representations and a unified network architecture, alleviating the CF issue without having to rely on replay data.

Synaptic intelligence (SI) is a regularization-based method [11] that mitigates CF by estimating how important each model parameter is for previously learned tasks [41]. During training, SI accumulates the contribution of each parameter to loss reduction by integrating the product of parameter updates and their corresponding gradients following a path-integral formulation. These accumulated scores are normalized to form importance weights that represent how crucial each parameter is for retaining past knowledge. When learning a new task, SI introduces a quadratic regularization term that penalizes changes to parameters with high importance while allowing less critical parameters to adapt freely. Unlike elastic weight consolidation (EWC) [27], SI performs this computation online without requiring additional passes over old data or storage of the Fisher matrix, leading to an efficient and memory-light mechanism for knowledge retention across sequential tasks.

Experience replay (ER) [15] is a replay-based CL method [11] which maintains a small memory buffer to store raw input images and the corresponding hard labels from previous tasks. When learning a new task, the model is trained on current task samples in combination with a small batch of stored samples from past tasks. By periodically revisiting examples from earlier tasks, ER preserves previously acquired knowledge through direct supervision to effectively mitigate the CF issue. ER leverages the reservoir sampling strategy [42] to ensure that every training example has an equal probability of being stored in the replay buffer, which prevents early-task bias. Under this mechanism, ER typically performs well as long as the memory capacity is sufficiently large and the replay coverage is adequate.

Dark experience replay (DER) [16] is another replay CL method [11]. DER extends ER by combining rehearsal with knowledge distillation, using both input samples and their stored logits to further mitigate forgetting. During training on each task, DER maintains a memory buffer of input images and their soft-target logits generated from the old model, then jointly optimizes the standard supervised loss on current data and a knowledge distillation loss on replayed samples that matches the model’s predictions to the stored logits of replayed samples. By preserving the prior model’s output distribution rather than relying only on hard labels, DER can retain richer learned information from old tasks and achieve more robust knowledge retention. DER++ is an extension of DER proposed by the same authors [16]. It provides an additional supervised loss on replayed memory images, integrating both hard-label replay and soft-logit distillation. This dual-objective design more effectively reinforces past knowledge and provides improved overall stability compared to DER. It also addresses the issue of highly biased logits resulting from a sudden distribution shift caused by the reservoir sampling method.

Dynamic token expansion (DyTox) is an architecture-based CL method specifically designed for the CIL scenario [17], with a transformer architecture that progresses when a new task is introduced to the model. DyTox separates feature extraction and task-specific adaptation using shared self-attention blocks (SAB) and task attention blocks (TAB). When a new task is introduced to the model, DyTox dynamically expands a set of task tokens and assigns a new classifier head while keeping the rest of the model shared. This dynamic expansion allows the model to learn new classes without forgetting previously learned knowledge. Rather than using a class token, as in ViT [43], DyToX adopts the idea from CaiT [44] of not using a class token in the beginning. This avoids the problem of the learned weights being used to optimize the two contradictory objectives of extracting features from patches and learning useful information for the learning classifier. This approach aims to better capture task-specific information while reducing optimization conflicts across tasks.

To alleviate the CF issue and encourage diversity among task tokens, DyTox introduces two auxiliary losses: the distillation loss, which prevents forgetting information from past tasks, and the divergence loss, which keeps the new task token distinct and expressive. This allows the model to find a good balance between plasticity and stability. Although DyTox achieves strong performance on natural image benchmarks, its architectural expansion strategy requires high-resolution input and training images to achieve strong performance on medical image datasets. As a result, its scalability to small-patch medical images remains limited, making it important to evaluate its transferability in real clinical domains such as histopathology.

## 4. Dataset Description

To verify the effectiveness of advanced CL methods on medical images, we utilize the NCT-CRC-HE-100K dataset, which consists of 100,000 non-overlapping H&E-stained image patches of human colorectal cancer and normal tissue [2]. The NCT-CRC-HE-100K dataset is one of the most widely used histopathology datasets for colorectal cancer research, supporting both tumor detection and multi-class colon tissue classification. It enables robust training and evaluation of deep neural networks on large-scale patch-level H&E images [45,46,47]. All images are provided in TIFF format at a resolution of 224×224 pixels with 8-bit depth.

**Figure 2:**
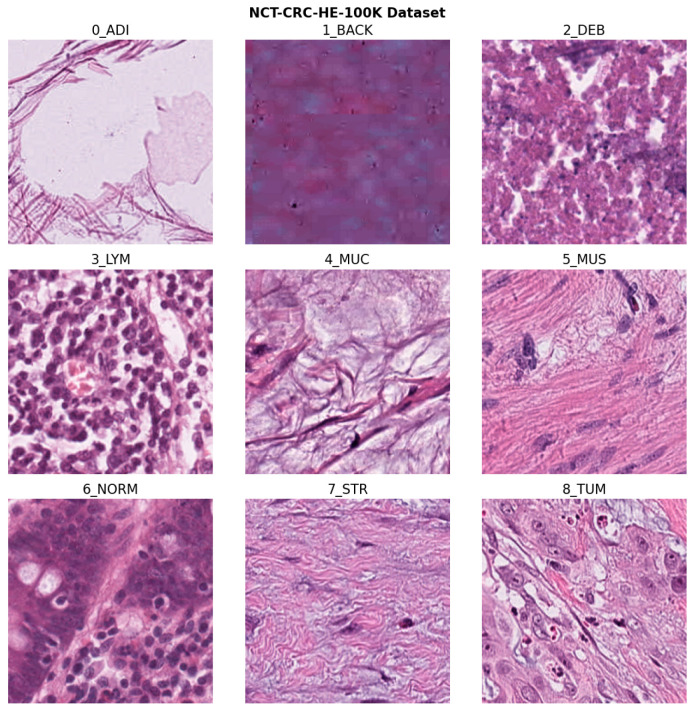
Histopathology images from NCT-CRC-HE-100K dataset.

Each patch is extracted from a whole-slide image and represents a localized tissue region such as tumor epithelium, healthy colon mucosa, connective tissue, immune cell infiltrates, adipose tissue, or background regions. The dataset contains histopathology image patches from nine tissue classes, including adipose tissue (ADI), background (BACK), normal colon mucosa (NORM), colorectal adenocarcinoma epithelium (TUM), and so on. To provide a clearer description of the dataset composition, the class distribution is reported in Table 1, where NCT-CRC-HE-100K exhibits a relatively uniform class distribution. Representative images are shown in Figure 2.

In addition to NCT-CRC-HE-100K, we also use CRC-HE-7K as an additional validation dataset to assess whether the main observations obtained from the primary benchmark can be generalized to a smaller independent colorectal histopathology dataset. CRC-HE-7K contains H&E-stained colorectal tissue image patches from the same tissue categories as NCT-CRC-HE-100K, but is smaller in scale. Therefore, NCT-CRC-HE-100K is used as the primary benchmark for the main experimental analyses reported in Section 6.1 and Section 6.2, while CRC-HE-7K is used for the additional generalization analysis reported in Section 6.3. The detailed construction of the CIL tasks and the experimental configuration are described in Section 5.

## 5. Experiment Design and Configuration

**Figure 3:**
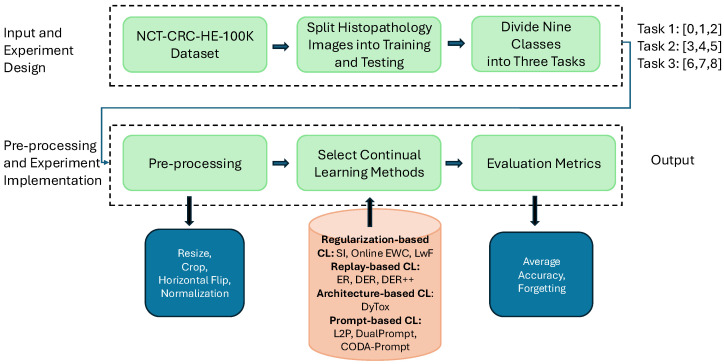
Experimental pipeline for continual learning on the NCT-CRC-HE-100K dataset.

We utilize ten different CL methods: three regularization-based methods (SI, Online EWC, and LwF) three replay-based methods (ER, DER, and DER++), one architecture-based method (DyTox), and three prompt-based methods (L2P, DualPrompt, and CODA-Prompt). All implementations are based on the Mammoth framework [48], which provides unified training pipelines, evaluation protocols, and memory buffer management for research purposes and has been widely adopted and validated in prior works [16,49,50,51]. Using Mammoth ensures correct, fair, and reproducible comparisons across methods. For DyTox, which is not currently included in Mammoth, we use the official implementation released by the authors, following the original training and evaluation settings. Figure 3 displays the overview of the experimental pipeline used to evaluate CL methods on the NCT-CRC-HE-100K histopathology dataset. This workflow includes preprocessing, task construction, selection of CL strategies (regularization-based, replay-based, architecture-based, and prompt-based), and evaluation metrics (average accuracy and forgetting).

We split the nine tissue categories into three sequential tasks according to their functional and diagnostic roles in colorectal cancer pathology, with each containing three classes. This setup is intended to approximate the staged expansion of a histopathology AI system, from basic tissue/background recognition to tissue-context characterization and tumor-related discrimination. Specifically, Task 1 includes ADI, BACK, and DEB, representing adipose tissue, background, and debris-related regions that are useful for filtering non-diagnostic or less diagnostically relevant areas [52]; Task 2 introduces LYM, MUC, and MUS, which correspond to lymphocyte-rich, mucinous, and smooth muscle regions that provide tissue-context information related to the tumor microenvironment [53]; finally, Task 3 introduces NORM, STR, and TUM, corresponding to normal mucosa, cancer-associated stroma, and tumor epithelium, which define the most clinically important diagnostic boundary in colorectal cancer pathology [52].

All methods are trained with a batch size of 64. A summary of the hyperparameters is provided in Table 2. All reported results are presented as mean ± standard deviation over three independent runs. All experiments were conducted on an NVIDIA GPU node equipped with NVIDIA A100-SXM4-80GB GPUs. Each experiment was run on a single NVIDIA A100-SXM4-80GB GPU with 80 GB memory. The experiments were performed using NVIDIA driver version 575.57.08 and CUDA version 12.9. The code developed for this study, including extensions to the Mammoth framework for dataset preparation, normalization, confusion matrices, ROC curves, misclassification analysis, loss and accuracy learning curves, training time measurement, and visualization, is publicly available at https://github.com/AILabLLL/CLMedical (accessed on 28 May 2026).

To study the best performance of these methods, we use the parameters suggested by their respective papers and modify them based on the characteristics of the different methods. First, for all prompt-based methods, we use the ViT-B/16 model pretrained on ImageNet-21k and fine-tuned on ImageNet-1k, as recommended for L2P, DualPrompt, and CODA-Prompt. Since the ViT backbone is a pretrained model, only a small number of training epochs is typically required for prompt-based methods. In practice, these methods usually train for 5, 10, or 20 epochs. For example, in the official implementation of L2P, the best performance is obtained with only 5 training epochs. Following this convention, we evaluate the methods within this small epoch range. For the learning rate, prompt-based models usually utilize a higher learning rate, such as 0.03 or 0.001 in the released code, as only the lightweight prompt modules are trained while the backbone remains frozen, making prompt optimization stable even under large learning rates.

For replay-based methods, we adopt ResNet-18 trained with the stochastic gradient descent (SGD) optimizer, following the original design choice and commonly used setting of DER [16]. This choice is made in order to evaluate each method family under the architectural setting for which it was originally designed. Specifically, the prompt-based methods introduced above are built upon frozen pretrained ViTs, where learnable prompt tokens are inserted into the input token sequence or selected transformer layers, as described in the original L2P, DualPrompt, and CODA-Prompt settings. Therefore, the mechanisms are closely coupled with the token-based architecture of ViT.

In contrast, replay-based methods such as ER, DER, and DER++ were originally developed and are commonly evaluated with CNN backbones, especially ResNet-style architectures trained with SGD. Accordingly, we use ViT-B/16 for prompt-based methods and ResNet-18 for replay-based methods in order to follow the canonical and method-faithful configurations of each CL method. The purpose of this setting is to report the representative performance of each method under its standard experimental configuration rather than to conduct a controlled comparison between the CNN and transformer backbones.

### 5.1. Data Normalization Strategies and Preprocessing Steps

Data normalization is a fundamental preprocessing step in computer vision that improves the stability, efficiency, and performance of neural networks. Four commonly used normalization strategies are ImageNet normalization, dataset-level normalization, per-image normalization, and Macenko stain normalization [26]. Some common computer vision tasks accept ImageNet normalization values, where

μ=[0.485,0.456,0.406],σ=[0.229,0.224,0.225]

However, because the data distribution of medical images, particularly histopathology images, differs from ImageNet images in color statistics, staining variability, and structural appearance, we decided to use a dataset-level normalization strategy to calculate the mean and standard deviation from our medical training dataset. This ensures consistent input distribution across samples, where

μ=[0.7408,0.5332,0.7060],σ=[0.1645,0.2173,0.1572]

Per-image normalization normalizes each image independently using its own mean and standard deviation, which reduces brightness and contrast variability across samples. Macenko stain normalization aligns the color appearance of H&E histopathology images by estimating and matching stain vectors in optical density space. To evaluate the effects of different normalization strategies, we compare two representative methods, DualPrompt and DER++, under the proposed dataset-level normalization versus other normalization strategies; for the experimental results, see Section 6.

In addition, we preprocess the histopathology images from the NCT-CRC-HE-100K dataset by first resizing them from 224×224 to 256×256 using bicubic interpolation, followed by random cropping back to 224×224 to introduce spatial variability and improve model generalization ability. Horizontal flipping is also applied as a standard data augmentation step. After preprocessing, we compute the dataset-specific mean and standard deviation for each RGB channel using the training images, then use these statistics to normalize the input data.

### 5.2. Evaluation Metrics

We evaluate CL performance in this work using two commonly adopted metrics, namely, average accuracy and forgetting. Average accuracy measures the average test accuracy across all tasks after training on the final task, reflecting the overall performance of the model throughout the task sequence. Forgetting quantifies the extent to which performance on previously learned tasks degrades after learning new tasks, measuring the model’s ability to retain prior knowledge. These metrics are both widely used in CL studies [16,20,21,22].

Higher average accuracy indicates that the model performs well across all tasks, whereas lower forgetting reflects stronger retention of previously learned knowledge. Therefore, an effective CL method is generally expected to achieve both high average accuracy and low forgetting. The metrics are defined as follow:

(6) $$Avg.Acc=\frac{1}{T}\sum_{i=1}^{T}A_{i,T}$$

(7) $$F=\frac{1}{T-1}\sum_{i=1}^{T-1}\left(max_{j\in [i,...,T]}A_{i,j}-A_{i,T}\right)$$

where *i* denotes the task index, *T* is total number of tasks (i.e., the final task), *j* denotes an intermediate task index between *i* and *T*, $A_{i,j}$ represents the test accuracy on task *i* after training up to task *j*, and $A_{i,T}$ denotes the test accuracy on task *i* after training on the final task *T*. Thus, average accuracy is computed as the mean performance across all tasks after training on the final task, while forgetting is computed as the average difference between the maximum accuracy achieved on each task during training and its final accuracy after learning all tasks.

## 6. Results Analysis

### 6.1. Overall Performance Comparison

In this section, we present the performance of different normalization strategies on histopathology images and compare ten CL approaches covering replay-based, prompt-based, architecture-based, and regularization-based methods. When presenting the results of replay-based and prompt-based methods, we also report the average accuracy and forgetting values under different buffer sizes and training epochs, allowing us to examine the influence of these hyperparameters on model performance.

To assess the effect of normalization strategies on the performance of the CL method, we evaluate two representative methods for DER++ and DualPrompt under four commonly used normalization types. Table 3 demonstrates the corresponding average accuracy results (epochs = 5 for DualPrompt; epochs = 50, buffer size = 500 for DER++). Based on the experimental results, it can be observed that dataset-level normalization consistently achieves the highest performance for both DER++ and DualPrompt, indicating that normalization aligned with the dataset’s inherent distribution is more effective than ImageNet-based or the other two normalization approaches.

The superior performance of dataset-level normalization can be explained by its better balance between domain alignment and preservation of discriminative histopathological cues. ImageNet normalization uses statistics derived from natural images, which may introduce a domain mismatch for H&E-stained histopathology patches. Per-image normalization standardizes each image independently, reducing image-level brightness and contrast variation but also suppressing differences in cross-sample color and staining intensity that may be informative for distinguishing tissue categories. Macenko normalization reduces stain variation by aligning images to a reference stain appearance, but may also remove useful stain-related differences or introduce additional variability when stain estimation is unstable.

In contrast, dataset-level normalization estimates the mean and standard deviation from the target training dataset and applies the same statistics to all samples. This provides a consistent histopathology-specific input scale while preserving relative differences in color and staining intensity across tissue categories. From the perspective of the stability–plasticity tradeoff in CIL, such consistent input scaling can reduce input-induced representation drift when new classes are introduced, thereby improving stability for previously learned classes while retaining sufficient plasticity to learn newly introduced tissue categories.

Table 4 and Table 5 further examine the influence of the training process by reporting the variation of average accuracy and forgetting under different training epoch settings. These results allow us to evaluate how training duration affects both overall performance and knowledge retention in the CIL setting for histopathology image classification, where new tissue categories are introduced sequentially across tasks (e.g., Task 1: [ADI, BACK, DEB], Task 2: [LYM, MUC, MUS], and Task 3: [NORM, STR, TUM]).

To examine how the buffer size and number of training epochs influence the effectiveness of replay-based methods, we evaluate ER, DER, and DER++ under different combinations of epoch (20, 50) and buffer size (200, 500). As shown in Table 4, DER and DER++ exhibit a clear trend in which increasing both the number of epochs and buffer size improves average accuracy while reducing forgetting, with the best performance observed at Epochs = 50 and Buffer size = 500. ER also benefits from larger buffers and longer training. Although ER achieves the highest average accuracy under certain settings, this is accompanied by substantially higher forgetting; in contrast, DER++ consistently attains the lowest forgetting while maintaining a competitive average accuracy. Thus, from a stability-oriented perspective, DER++ is preferable among the replay-based methods, as it better preserves previously learned tissue-category knowledge while learning new classes.

These observations also highlight a limitation in the current approach to evaluating CL methods. When comparing different methods, average accuracy and forgetting may favor different models; one method may achieve higher accuracy but suffer from greater forgetting, whereas another may obtain slightly lower accuracy while preserving previous knowledge more effectively. Although composite evaluation schemes have been proposed, including CLscore, which combines criteria including accuracy, backward transfer, memory usage, and computational efficiency [54], these metrics are designed for broader multi-criteria evaluation and may depend on application-specific weighting. In our setting, the key challenge is the lack of a simple and interpretable unified metric for jointly considering average accuracy and forgetting when selecting the best-performing CL method. Developing such a metric remains an important direction for future work.

To examine whether prompt-based methods require only a small number of epochs to achieve good generalization, we evaluate L2P, CODA-Prompt, and DualPrompt using 5, 10, and 20 training epochs. As shown in Table 5, L2P and DualPrompt achieve their best performance on the NCT-CRC-HE-100K dataset at 5 epochs, indicating that these methods converge effectively under a short training schedule. In contrast, CODA-Prompt continues to improve with longer training and achieves better performance at 20 epochs. These results demonstrate that the effect of training duration differs across prompt-based methods.

Overall, DER++ and DualPrompt are the leading methods within the replay-based and prompt-based categories, respectively, under our experimental settings. Our analysis of sensitivity to the number of epochs further shows that L2P and DualPrompt reach their best performance at 5 epochs, while CODA-Prompt continues to improve with longer training. These findings motivate a more detailed analysis of the tradeoffs between replay-based and prompt-based strategies in terms of performance, efficiency, stability, and deployment feasibility.

**Figure 4:**
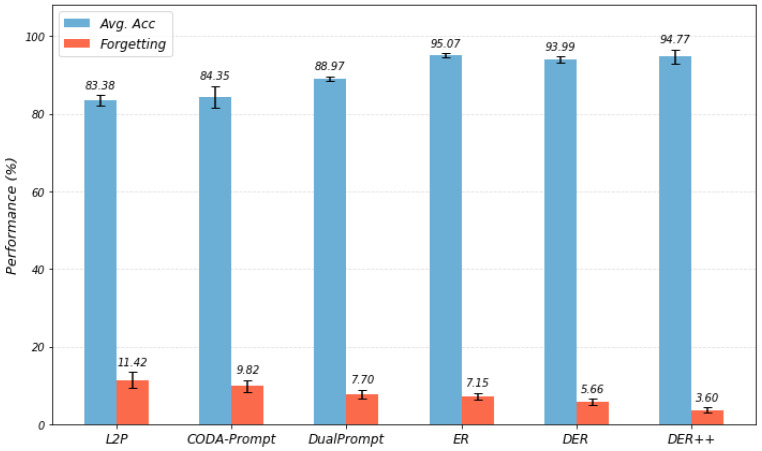
Comparison of prompt-based and replay-based methods.

Figure 4 shows that replay-based CL methods (ER, DER, and DER++) achieve higher average accuracy and lower forgetting than prompt-based methods (L2P, CODA-Prompt, and DualPrompt) when sufficient replay buffer and training epochs are available. However, these gains require longer training and come at the cost of storing past samples. In contrast, DualPrompt achieves competitive performance while training for only 5 epochs and without replaying past data. Therefore, in privacy-constrained clinical environments, prompt-based methods provide a practical and effective alternative for maintaining performance on both newly introduced and previously learned tasks. This is particularly relevant for the histopathology image classification task addressed in this work.

On the NCT class-incremental benchmark, regularization-based methods such as Online EWC, LwF, and SI still exhibit substantial catastrophic forgetting even after extensive hyperparameter tuning. Increasing the regularization strength to favor previously acquired knowledge (e.g., λ=50 for Online EWC, α=2.0 for LwF, and c=2.0 for SI) results in reduced plasticity but does not prevent performance degradation, with models still showing severe forgetting of earlier tasks. As reported in Table 6, these methods achieve very low average accuracy (16–30%) with severe forgetting (75–100%) and with noticeable variance across different random seeds, indicating instability under the class-incremental histopathology setting. These results suggest that parameter-space regularization alone is insufficient to handle the strong distribution shift and class interference inherent to CIL. This behavior aligns with prior studies indicating that regularization-based methods often fail in CIL settings because they lack positive training signals for previously learned classes [55]. Furthermore, under the single-head evaluation setup, these methods suffer from severe class imbalance and logit bias towards new classes, leading to catastrophic forgetting [16,56]. Consequently, our results confirm the limitations of regularization-based approaches in realistic medical scenarios with expanding diagnostic categories.

For completeness, we also evaluate DyTox on the NCT-CRC-HE-100K dataset for a more comprehensive comparison. Under 500 training epochs with a memory buffer of 1000, DyTox achieves an average accuracy of 25.09 and a forgetting score of 8.59 on our histopathology classification tasks. These results indicate that DyTox struggles to learn discriminative representations for histopathology images in the CIL setting. By jointly examining accuracy and forgetting, we observe that this limitation is primarily reflected in low final accuracy rather than excessive forgetting; while DyTox exhibits comparable forgetting to other methods, its accuracy remains substantially lower. This behavior can be explained by the interaction between DyTox’s architecture based on shared tokens and the fine-grained characteristics of colorectal histopathology patches.

From the architectural perspective, DyTox relies on shared self-attention encoder blocks to extract patch token representations across all tasks, while task-specific information is mainly modeled through dynamically expanded task tokens and classifier branches [17]. Therefore, the effectiveness of the task-specific tokens depends strongly on whether the shared encoder has already preserved discriminative pathological features. This design differs from replay-based methods [31], which can revisit previous samples to reinforce old class-specific tissue patterns, as well as from prompt-based methods such as DualPrompt [21], which exploit a pretrained ViT backbone and use general and expert prompts to guide task-adaptive feature extraction. In contrast, DyTox updates the shared transformer representation during incremental learning, and does not explicitly preserve previous histopathology patterns through replay or a frozen pretrained representation space.

This design is less favorable for colorectal histopathology images, where discriminative information often appears as subtle, local, and spatially-distributed microtextures rather than as object-level semantic structures [9,57]. Tissue categories such as tumor epithelium, normal mucosa, mucus, debris, stroma, glandular organization, staining variation, and local epithelial arrangement can be visually similar within 224×224 patches [52]. Fixed-size tokenization may weaken or merge these local pathological cues before they reach the task token decoder. As a result, when visually similar tissue classes are introduced incrementally, the shared representation may drift and the expanded task tokens may be insufficient to preserve old class-specific microtexture patterns. This explains why DyTox performs poorly in our experiments despite its architecture expansion-based design.

These results indicate clear differences in how CL paradigms behave on histopathology images, motivating further analysis of their underlying mechanisms and related practical implications.

### 6.2. Clinical Relevance and Error Analysis

#### 6.2.1. Diagnostic Performance Evaluation

**Figure 5:**
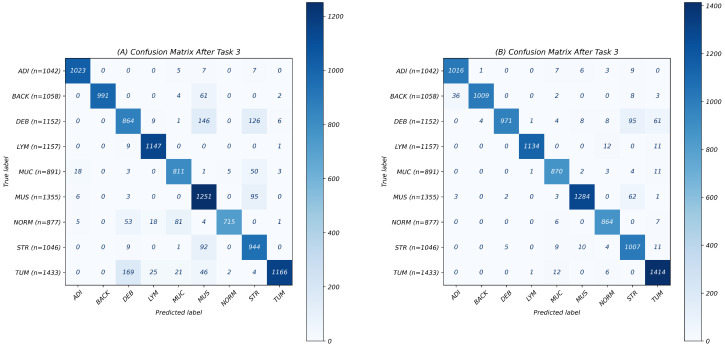
Confusion matrices of representative continual learning methods on the NCT-CRC-HE-100K dataset. (**A**) Confusion Matrix of DualPrompt. (**B**) Confusion Matrix of DER++.

We further analyze the clinical relevance of representative CL methods by examining class-wise diagnostic behavior using confusion matrices, multiclass ROC curves, and class-wise precision, recall, and F1 scores for DualPrompt and DER++ on the NCT-CRC-HE-100K dataset. These analyses evaluate performance across all learned tissue classes after completion of Task 3. The diagonal entries in Figure 5 represent the true positive counts for each tissue class; the strong diagonal dominance indicates that most samples are correctly assigned to their corresponding categories. Among these tissue categories, the separation between NORM and TUM is clinically important because it reflects the model’s ability to distinguish normal mucosa from malignant epithelial tissue [2].

Both DualPrompt and DER++ show limited direct confusion between NORM and TUM, suggesting that they preserve a clear distinction between normal mucosa and tumor epithelium after Task 3. DualPrompt produces slightly fewer direct cross-confusions, with 1/877 NORM samples misclassified as TUM (0.11%) and 2/1433 TUM samples misclassified as NORM (0.14%), compared with 7/877 (0.80%) and 6/1433 (0.42%) for DER++, respectively. In our CIL setting, NORM, STR, and TUM are introduced together in Task 3, which may help the model to learn fine-grained morphological differences among these clinically related tissue classes within the same training context. Nevertheless, DER++ achieves stronger overall class-wise performance, especially for TUM, indicating that the two methods exhibit complementary strengths.

Beyond the (NORM, TUM) pair, the main off-diagonal errors occur among visually related tissue components. DualPrompt shows relatively higher confusion involving DEB, MUS, STR, and TUM, such as DEB predicted as MUS or STR and TUM predicted as DEB. In contrast, DER++ reduces most overall off-diagonal errors and achieves stronger class-wise recognition, particularly for TUM, where 1414/1433 samples are correctly classified, compared with 1166/1433 for DualPrompt. DER++ also improves DEB recognition, correctly classifying 971/1152 DEB samples, compared with 864/1152 for DualPrompt. These results suggest that replay-based consolidation can provide more stable class-wise representations in histopathological CIL, while DualPrompt maintains lower direct (NORM, TUM) cross-confusion without storing exemplars.

**Figure 6:**
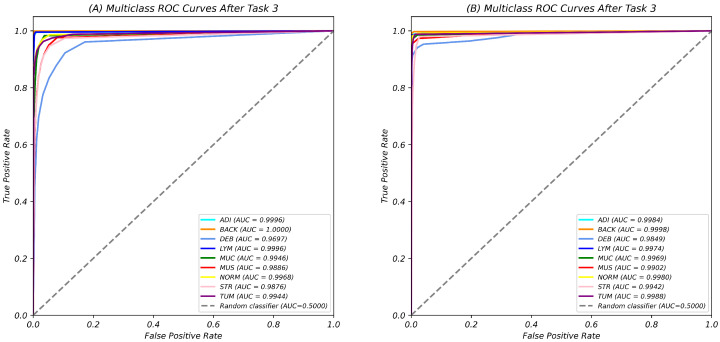
ROC Curves of representative continual learning methods on the NCT-CRC-HE-100K dataset. (**A**) ROC Curve of DualPrompt. (**B**) ROC Curve of DER++.

The ROC curves in Figure 6 provide a complementary probability-based evaluation of class separability. The area under the ROC curve (AUC) evaluates ranking-based discriminative ability under different decision thresholds [58,59]. In this study, AUC is calculated from the predicted probabilities obtained from the softmax output in a one-vs-rest manner; therefore, high AUC values may coexist with off-diagonal errors in the confusion matrix when the correct class still receives higher confidence scores than most negative classes. Both methods achieve high AUC values across all tissue categories, indicating strong probabilistic separability. DER++ obtains consistently high AUC values, including 0.9849 for DEB and 0.9988 for TUM, while DualPrompt achieves comparable AUC values of 0.9697 and 0.9944 for the same classes, respectively. These results suggest that replay-based methods such as DER++ can provide stronger class-wise stability when memory replay is available, whereas prompt-based methods such as DualPrompt still offer competitive discrimination without storing exemplars. Overall, the confusion matrix and ROC analyses demonstrate that both methods provide diagnostically relevant class-wise discrimination on the NCT-CRC-HE-100K dataset, supporting the complementary benefits of replay-based and prompt-based CL strategies in histopathology image classification.

To complement the confusion matrix analysis with quantitative class-wise metrics, Table 7 reports the class-wise performance of DualPrompt and DER++ on the histopathology image dataset (NCT-CRC-HE-100K). Overall, DER++ achieves stronger performance than DualPrompt on several classes, particularly for clinically important categories such as NORM and TUM. For these two classes, DER++ obtains F1 scores of 0.97 and 0.96, respectively, whereas DualPrompt achieves 0.89 for both categories. This indicates that replay-based learning remains advantageous for preserving discriminative features in diagnostically relevant tissue classes. However, DER++ relies on storing and replaying exemplars from previous tasks, which may be impractical or restricted in privacy-sensitive medical imaging scenarios. In contrast, DualPrompt is exemplar-free, allowing it to avoid retention of historical patient images. Despite its lower performance on NORM and TUM, DualPrompt still achieves reasonably high F1 scores for these classes and remains competitive on ADI, BACK, and LYM. These results suggest that exemplar-free methods provide a promising privacy-preserving alternative, although further improvement is needed to close the performance gap on clinically critical classes.

#### 6.2.2. Misclassification Analysis

**Figure 7:**
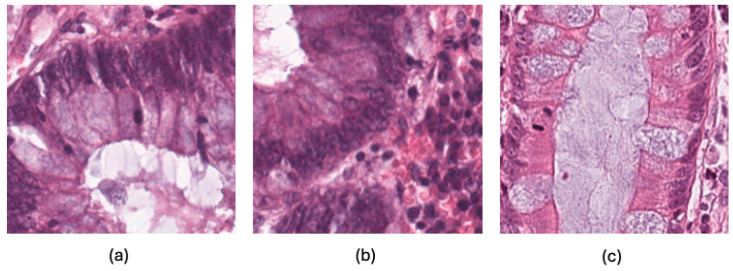
Representative misclassified cases produced by DualPrompt on the NCT-CRC-HE-100K dataset: (**a**) Case 1: TUM predicted as NORM; (**b**) Case 2: TUM predicted as NORM; (**c**) Case 3: NORM predicted as TUM. Predicted class probabilities are reported below each patch.

Beyond aggregate metrics, the clinical significance of a CL classification model depends on the nature and distribution of its errors. We present a preliminary analysis of three representative misclassification cases involving the clinically most critical confusion boundary, namely, NORM-to-TUM and TUM-to-NORM, produced by DualPrompt on the NCT-CRC-HE-100K dataset. These cases are of particular interest because, despite its lower overall accuracy, DualPrompt produces substantially fewer NORM˘2194TUM confusions than DER++ (3 vs. 13 cases, a 76.9% reduction), suggesting a qualitatively different and more clinically favorable error distribution at this boundary. The three cases illustrated in Figure 7 are selected in order to represent distinct failure mechanisms with differing clinical implications: Cases (a) and (b) are false negatives in which TUM patches are misclassified as NORM, while Case (c) is a false positive in which a NORM patch is misclassified as TUM. The following case analyses are based on model output probabilities and visual inspection of patch appearance.

Case (a)—Decision Boundary Ambiguity (TUM predicted as NORM). DualPrompt assigns NORM = 38.67%, MUC = 31.65%, and TUM = 25.28% to a tumor patch, with the ground-truth class receiving only 25.28%. The probability mass is distributed nearly uniformly across three classes. This output pattern indicates that the model fails to assign a confident prediction to this sample. From a deployment perspective, this is the most manageable type of error; the low maximum confidence (38.67%) falls well below any reasonable deployment threshold, meaning that an uncertainty-aware model could automatically defer this case to pathologist review rather than issuing an automated prediction. Visually, the patch contains a large acellular region with pale staining and surrounding epithelial structures with a regular tubular arrangement. At the 224×224 patch scale and without broader tissue-level context, the visual features of this patch appear to share characteristics with more than one class label as defined in the NCT-CRC-HE-100K annotation protocol [52], which may explain why the model distributes the probability across multiple classes rather than concentrating it on TUM. From a deployment perspective, this is the most manageable error type: a maximum class probability of 38.67% falls well below any reasonable confidence threshold, meaning that an uncertainty-aware system could automatically defer this case to pathologist review [60]. The underlying visual or feature-level causes cannot be determined from model outputs alone, and would require expert validation to confirm.

Case (b)—Tissue quality degradation (TUM predicted as NORM). DualPrompt assigns NORM = 32.94%, DEB = 28.77%, LYM = 14.78%, and TUM = 9.57%, with the ground-truth class ranking fifth. Unlike Case (a), the probability mass is spread across four classes. Visually, the patch contains regions of densely-packed cells with dark staining alongside areas of pale structureless material. These non-TUM visual components appear to occupy a substantial proportion of the patch’s pixel area, suggesting that the model’s prediction is primarily driven by non-TUM visual features. This is consistent with the known susceptibility of exemplar-free CL methods to distribution shifts arising from variability in tissue preparation quality and necrosis levels across patient samples, and is further supported by DualPrompt’s low DEB F1 score of 0.76 (vs. 0.91 for DER++, Table 7). The gap between the top two predictions is only 4.17 percentage points (NORM 32.94% vs. DEB 28.77%), indicating that the model is nearly equally uncertain between these two classes. The pale structureless regions of the patch can share visual characteristics with both the DEB category (cellular debris, low structure) and the NORM category (pale glandular lumen regions) defined in the NCT-CRC-HE-100K annotation protocol [52]. This overlap may cause the model to marginally favor NORM over DEB, despite neither class receiving strong activation. We note that this is an observational interpretation based on model output probabilities and patch appearance.

Case (c)—High-confidence misclassification (NORM predicted as TUM). DualPrompt assigns TUM = 54.98%, MUC = 32.20%, and NORM = 0.06% to a patch labeled NORM. In contrast to Cases (a) and (b), the model expresses high confidence in a single incorrect class, with effectively zero probability assigned to the ground-truth label. Rather than being near the decision boundary, this sample receives a prediction that is both decisive and wrong. Visually, the patch contains regular tubular structures along with a large central lumen region featuring pale staining and an organized cellular lining. The pale lumen region visually resembles tissue categories associated with mucinous content in the NCT-CRC-HE-100K dataset [52], which may partially explain the elevated probability of MUC (32.20%). The densely-arranged cellular regions near the patch margin may share visual characteristics with TUM-labeled patches in the training set, potentially driving the high TUM prediction. The critical deployment implication is that this error, unlike Cases (a) and (b), cannot be detected or mitigated by any confidence threshold mechanism, as the model assigns 54.98% to the incorrect class with near-zero residual uncertainty. Such errors would represent a high-risk failure mode if the model were incorporated into a clinical screening pipeline, since they might not be easily detected by simple confidence threshold-based mechanisms.

### 6.3. Generalization Analysis

To further examine whether our observations generalize beyond the NCT-CRC-HE-100K dataset, we conduct additional experiments on the CRC-HE-7K dataset using the representative CL categories (prompt-based methods and replay-based methods). The CRC-HE-7K dataset [2] contains 7180 histopathological images collected from 50 patients with colorectal adenocarcinoma, and has no overlapping images with the NCT-CRC-HE-100K dataset.

Table 8 shows that the dataset-level normalization strategy also improves performance on the CRC-HE-7K dataset. In particular, both DER++ and DualPrompt achieve their best results under dataset normalization compared with the other normalization strategies, suggesting that dataset-level statistics provide a more suitable normalization scheme for this histopathology dataset.

Based on the results in Table 9 and Table 10, we observe trends on the CRC-HE-7K dataset that are consistent with those obtained on the NCT-CRC-HE-100K dataset. First, when considering the stability–plasticity tradeoff as measured by forgetting and average accuracy, DER++ performs better than ER and DER among replay-based methods, while DualPrompt outperforms L2P and CODA-Prompt among prompt-based methods. Second, for replay-based methods, increasing the number of training epochs and buffer size generally improves the performance of ER and DER++. In contrast, DER achieves its best performance with 20 epochs and a buffer size of 200, which may be attributed to the distribution of replay samples. Similar to the observations on the NCT-CRC-HE-100K dataset, Table 10 shows that L2P and DualPrompt achieve their best balance between high average accuracy and low forgetting with 5 training epochs. For CODA-Prompt, however, better performance is obtained with more training epochs, likely because its more complex architecture and larger number of parameters require additional training steps for effective optimization. Although increasing the number of epochs may slightly improve the overall average accuracy for the DualPrompt method, it also increases forgetting; therefore, we used the setting with 5 epochs to achieve a better balance between classification performance and forgetting control.

The CRC-HE-7K results show that the main trends observed on the NCT-CRC-HE-100K dataset are largely preserved. First, the effect of normalization remains consistent, with dataset-level normalization achieving the best average accuracy for both DER++ and DualPrompt among the evaluated normalization strategies. This suggests that the benefit of dataset-level normalization is not restricted to the original NCT-CRC-HE-100K split. Second, the relative behavior within CL method families is also consistent. Among replay-based methods, DER++ remains a strong baseline and achieves the best average accuracy and lowest forgetting under the setting with 50 epochs and a buffer size of 500. Among prompt-based methods, DualPrompt shows the strongest and most stable performance, achieving high average accuracy with low forgetting across training epochs. These findings provide additional support for our conclusion that DualPrompt can serve as a competitive replay-free alternative to replay-based methods in colorectal histopathology CIL.

**Figure 8:**
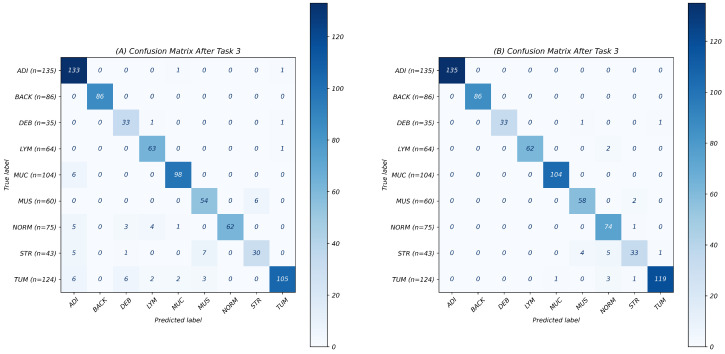
Confusion matrices of representative continual learning methods on the CRC-HE-7K dataset. (**A**) Confusion Matrix of DualPrompt. (**B**) Confusion Matrix of DER++.

Figure 8 displays the confusion matrices of DualPrompt and DER++ on the CRC-HE-7K dataset. Both methods achieve strong diagonal dominance, indicating that the selected CL methods maintain high class-wise classification accuracy on the additional dataset. DER++ shows fewer off-diagonal errors than DualPrompt, suggesting better generalization and more stable performance across histological classes. In particular, DualPrompt does not misclassify NORM samples as TUM, which is clinically meaningful for reducing false tumor predictions.

**Figure 9:**
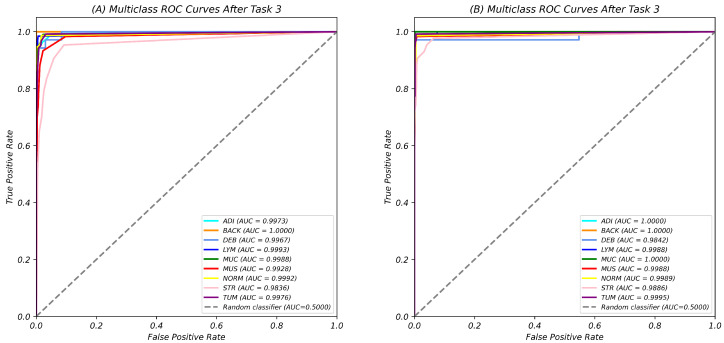
ROC Curves of representative continual learning methods on the CRC-HE-7K dataset. (**A**) ROC Curve of DualPrompt. (**B**) ROC Curve of DER++.

Figure 9 presents the multiclass ROC curves of DualPrompt and DER++ on CRC-HE-7K. Both methods obtain high AUC values across most classes, demonstrating strong discriminative capability on the additional dataset. These results further support the conclusion that the selected prompt-based and replay-based CL methods generalize well beyond the NCT-CRC-HE-100K dataset.

### 6.4. Statistical Significance Analysis

To evaluate the performance differences between DualPrompt and DER++, which are the top-performing methods in their respective CL families, we conduct an independent two-sample *t*-test comparing their final average accuracy results across three independent runs. DER++ achieves a mean average accuracy of 94.77%, while DualPrompt achieves 88.97%. The analysis yields a t-statistic of 4.14 and *p*-value of 0.0536. Since this *p*-value is slightly above the conventional 0.05 threshold, the difference is not statistically significant at the 0.05 level; however, the results do suggest a trend towards a performance difference between the two methods. We note that this statistical analysis is limited by the small number of independent runs, which reduces the statistical power of the test. Increasing the number of runs would provide a more reliable estimate of statistical significance; however, each full experiment requires substantial GPU computation time.

Beyond this statistical comparison, we provide additional supplementary analyses of training dynamics, forgetting behavior, overfitting tendencies, and training time in the Supplementary Materials to further support the interpretation of the overall results.

## 7. Discussion

Overall, our experiments show that the regularization-based methods demonstrate noticeably lower performance than the replay-based and prompt-based techniques. This is consistent with prior CL studies showing that traditional regularization-based approaches often struggle on modern CL benchmarks, suggesting that they may be less suitable for complex histopathology CIL tasks. In contrast, the replay-based and prompt-based methods achieve stronger overall performance, although they present different tradeoffs in terms of accuracy, forgetting, efficiency, training stability, privacy considerations, and deployment feasibility.

Another important finding is that the normalization strategy plays a substantial role in applying CL to histopathology images. In particular, dataset-level normalization consistently outperforms other normalization strategies. This suggests that aligning data preprocessing with the underlying distribution of medical images is important for achieving stable and robust CL performance. Since histopathology images exhibit stain variability and domain-specific intensity distributions that differ substantially from natural images, appropriate normalization is especially important when adapting CL methods originally developed for natural image benchmarks.

Following the comparison of different CL method families, we further examine whether their training duration is sufficient. In our experimental results, L2P and DualPrompt achieve their best performance with 5 epochs, suggesting that a short training schedule is sufficient for these two methods under our experimental setting. This finding is consistent with their parameter-efficient design, in which the pretrained ViT backbone remains frozen and only lightweight prompt parameters and the classifier are optimized. Importantly, we explicitly evaluated 5, 10, and 20 epochs for longer training. As shown in Table 5, increasing the number of epochs does not further improve L2P or DualPrompt, and DualPrompt even shows reduced performance with longer training. In contrast, CODA-Prompt benefits from longer training and achieves better performance with 20 epochs, likely because its attention-based prompt composition mechanism introduces additional prompt-related optimization components. These findings suggest that prompt-based CL methods should be evaluated using epoch sensitivity rather than assuming a fixed training budget across different prompt designs.

From a deployment perspective, DER++ relies on storing previous histopathology samples for replay, which may introduce privacy, data governance, and regulatory considerations in clinical settings [61,62,63,64]. In contrast, DualPrompt avoids raw-sample replay and provides competitive performance with lower memory cost, shorter training time, smoother training dynamics, and reduced privacy risk, as shown in Table 11. Therefore, although DER++ serves as a strong replay-based baseline when replay storage is permitted, DualPrompt provides a practical prompt-based alternative for privacy- and resource-constrained histopathology CIL deployment.

Compared with the existing medical imaging CL literature, our work offers a more comprehensive and clinically oriented evaluation framework, covering dataset characteristics, modality, image size, CL scenario, normalization analysis, and evaluated CL methods along with additional evaluation dimensions, as summarized in Table 12. Since prior studies differ in these aspects, the comparison is not intended as a direct numerical ranking; instead, it provides a contextual overview of how our study complements and extends prior medical CL work.

In this context, our work provides a broad benchmark for histopathology CIL. Specifically, we evaluate multiple CL paradigms, including regularization-based, architecture-based, replay-based, and prompt-based methods, on patch-level colorectal histopathology images at 224×224 resolution, whereas several prior medical CL evaluations focus on low-resolution MedMNIST images at 28×28 resolution. Beyond standard performance metrics, we further analyze normalization strategies, training dynamics, and computational efficiency. We also include clinically relevant evaluations using confusion matrices, ROC curves, and representative misclassification cases while also analyzing statistical significance to assess the robustness of the observed differences. Together, these analyses distinguish our study from existing medical CL benchmarks and provide a detailed understanding of model behavior, computational requirements, and practical considerations in histopathology CIL.

Despite these contributions, this study has several limitations. Our experiments are limited to two patch-level colorectal histopathology datasets, and broader validation across additional organs, institutions, staining protocols, and whole-slide image settings remains needed. In addition, CL evaluation lacks a unified metric that directly summarizes the tradeoff between average accuracy and forgetting. Finally, although we include clinically relevant analyses using confusion matrices, ROC curves, and representative misclassification cases, future work should incorporate physician or pathologist review in order to better assess clinical relevance and model failure modes.

## 8. Conclusions

In this study, we have presented a comprehensive benchmark for colorectal histopathology CIL. We evaluate representative regularization-based, architecture-based, replay-based, and prompt-based CL methods on the NCT-CRC-HE-100K dataset, with additional validation on CRC-HE-7K. Our results show that replay-based and prompt-based methods generally outperform regularization-based and and architecture-based ones, while dataset-level normalization consistently improves CL performance in histopathology images.

Beyond average accuracy and forgetting, we further analyze clinical-oriented evaluation, training dynamics, efficiency, and deployment feasibility. DER++ achieves strong performance among the evaluated replay-based methods in our experiments, but requires substantially higher GPU memory, longer training times, and storage of previous histopathology samples. It also exhibits more fluctuating training dynamics, and its reliance on replay buffers may introduce additional concerns around privacy and data governance in clinical deployment. By comparison, DualPrompt provides a competitive exemplar-free alternative in our histopathology CIL setting along with with lower computational cost, smoother training behavior, and reduced privacy-related data storage concerns, highlighting its potential practical value for privacy-constrained histopathology CIL contexts.

Future work may proceed in two directions. In the near term, further studies could improve prompt-based CL methods for histopathology CIL and evaluate their effectiveness across broader external cohorts, including additional institutions, organs, staining protocols, and whole-slide image workflows. In the longer term, future research could develop simple and interpretable unified metrics for summarizing the tradeoff between average accuracy and forgetting; additionally, privacy-preserving federated settings could be explored in which multiple institutions collaboratively adapt models to evolving data distributions without sharing raw patient images.

## Figures and Tables

**Table 1:** Class distribution of the NCT-CRC-HE-100K dataset.

Class ID	Class	Tissue Type	Number of Images
0	ADI	Adipose tissue	10,407
1	BACK	Background	10,566
2	DEB	Debris	11,512
3	LYM	Lymphocytes	11,557
4	MUC	Mucus	8896
5	MUS	Smooth muscle	13,536
6	NORM	Normal colon mucosa	8763
7	STR	Cancer-associated stroma	10,446
8	TUM	Colorectal adenocarcinoma epithelium	14,317
Total	–	–	100,000

**Table 2:** Training hyperparameters for different CL methods.

Method	Backbone	Optimizer	Learning Rate
ER/DER/DER++	ResNet-18	SGD	0.03
DyTox	convit	Adam	0.0005
L2P/DualPrompt	ViT-B/16 (frozen)	Adam	0.03
CODA-Prompt	ViT-B/16 (frozen)	Adam	0.001
Online EWC/LwF/SI	ResNet-18	SGD	0.001

**Table 3:** Effect of normalization type on the performance of representative CL methods on the NCT-CRC-HE-100K dataset.

CL Method	Macenko	Per-Image	ImageNet Norm	Dataset Norm
DER++	73.95 ± 10.11	92.88 ± 1.83	53.28 ± 37.08	94.77 ± 1.82
DualPrompt	65.72 ± 2.27	79.53 ± 2.52	86.25 ± 1.74	88.97 ± 0.60

**Table 4:** Effect of buffer size and training epochs on the performance of replay-based CL methods on the NCT-CRC-HE-100K dataset.

Method	Epoch	Buffer Size	Avg. Acc	Forgetting
ER	20	200	68.69 ± 16.83	32.37 ± 13.18
	20	500	73.67 ± 25.95	29.21 ± 27.44
	50	200	87.28 ± 2.81	16.20 ± 7.16
	50	500	95.07 ± 0.50	7.15 ± 0.72
DER	20	200	48.74 ± 35.34	52.33 ± 42.50
	20	500	92.79 ± 2.38	8.04 ± 4.35
	50	200	82.44 ± 11.94	22.26 ± 17.03
	50	500	93.99 ± 0.74	5.66 ± 2.53
DER++	20	200	73.52 ± 28.31	28.65 ± 32.10
	20	500	75.70 ± 27.65	26.87 ± 32.04
	50	200	90.85 ± 1.14	9.67 ± 3.25
	50	500	94.77 ± 1.82	3.66 ± 1.73

**Table 5:** Effect of training epochs on the performance of prompt-based CL methods on the NCT-CRC-HE-100K dataset.

Method	Epoch	Avg. Acc	Forgetting
L2P	5	83.38 ± 1.39	11.42 ± 1.97
	10	79.67 ± 4.21	19.95 ± 5.30
	20	82.92 ± 2.51	14.80 ± 3.47
CODA-Prompt	5	82.28 ± 2.63	12.40 ± 3.52
	10	82.04 ± 3.26	14.89 ± 5.47
	20	84.35 ± 2.75	9.82 ± 1.51
DualPrompt	5	88.97 ± 0.60	7.70 ± 1.21
	10	88.50 ± 1.14	9.46 ± 1.27
	20	87.01 ± 1.33	7.95 ± 2.62

**Table 6:** Performance of regularization-based CL methods.

Method	Avg. Acc	Forgetting
Online EWC	29.62 ± 4.83	74.51 ± 8.50
LwF	16.88 ± 13.03	99.77 ± 0.23
SI	29.66 ± 5.00	94.21 ± 9.82

**Table 7:** Performance comparison of representative continual learning methods for histopathology image datasets.

Class	DualPrompt			DER++		
	Precision	Recall	F1 Score	Precision	Recall	F1 Score
ADI	0.98	0.97	0.98	0.98	0.96	0.97
BACK	0.94	1.00	0.97	0.95	1.00	0.97
DEB	0.75	0.78	0.76	0.84	0.99	0.91
LYM	0.99	0.96	0.97	0.98	1.00	0.99
MUC	0.91	0.88	0.89	0.98	0.95	0.96
MUS	0.92	0.78	0.84	0.95	0.98	0.96
NORM	0.82	0.99	0.89	0.99	0.96	0.97
STR	0.90	0.77	0.83	0.96	0.85	0.90
TUM	0.81	0.99	0.89	0.99	0.93	0.96

**Table 8:** Effect of normalization type on the performance of representative CL methods on the CRC-HE-7K dataset.

CL Method	Macenko	Per-Image	ImageNet Norm	Dataset Norm
DER++	62.16 ± 9.74	93.16 ± 1.58	94.85 ± 0.84	95.15 ± 1.56
DualPrompt	75.27 ± 0.83	89.21 ± 0.41	82.43 ± 2.40	90.99 ± 0.33

**Table 9:** Effect of buffer size and training epochs on the performance of replay-based CL methods on the CRC-HE-7K dataset.

Method	Epoch	Buffer Size	Avg. Acc	Forgetting
ER	20	200	93.55 ± 2.01	6.50 ± 4.66
	20	500	93.62 ± 2.81	5.76 ± 2.79
	50	200	95.67 ± 0.28	5.32 ± 0.31
	50	500	95.33 ± 2.06	2.58 ± 0.25
DER	20	200	89.64 ± 1.70	8.11 ± 2.16
	20	500	74.18 ± 15.22	32.94 ± 22.09
	50	200	88.73 ± 3.15	12.28 ± 0.75
	50	500	88.68 ± 5.87	12.45 ± 8.23
DER++	20	200	91.37 ± 1.34	3.87 ± 2.29
	20	500	93.37 ± 1.20	2.75 ± 1.54
	50	200	92.71 ± 3.63	4.15 ± 2.15
	50	500	95.15 ± 1.56	1.77 ± 1.62

**Table 10:** Effect of training epochs on the performance of prompt-based CL methods on the CRC-HE-7K dataset.

Method	Epoch	Avg. Acc	Forgetting
L2P	5	86.79 ± 1.05	6.04 ± 1.61
	10	78.19 ± 2.54	25.32 ± 8.55
	20	80.14 ± 6.49	24.74 ± 8.55
CODA-Prompt	5	85.00 ± 0.78	4.85 ± 0.63
	10	86.22 ± 4.41	8.84 ± 3.87
	20	87.77 ± 1.45	10.29 ± 2.55
DualPrompt	5	90.99 ± 0.33	1.53 ± 0.88
	10	92.24 ± 0.77	2.71 ± 0.87
	20	92.26 ± 0.56	3.97 ± 1.60

**Table 11:** Comparison of performance and deployment tradeoffs between DualPrompt and DER++ on NCT-CRC-HE-100K.

Method	CL Paradigm	Avg. Acc	Forgetting	GPU Memory	Training Time	Training Stability	Privacy Concern
DualPrompt	Prompt-base	88.97	7.70	7570MiB	00:55:09	Smooth	Low
DER++	Replay-based	94.77 (+5.80)	3.66 (−4.04)	53182MiB (7.03×)	17:43:53 (19.29×)	Fluctuating	High

**Table 12:** Comparison of representative continual learning studies in medical image classification. TIL: task-incremental learning [30]; DIL: domain-incremental learning [30]; Cross-DIL: cross-domain incremental learning [31]; Norm. Study: whether the study systematically evaluates different input normalization strategies; CREA: clinical relevance and error analysis, including confusion matrix, ROC curve, and misclassification analyses; Training Dynamics: loss and accuracy curves; Efficiency Analysis: training time analysis and GPU memory usage; Mixed: the multiple medical imaging modalities of the MedMNIST dataset.

Literature	Dataset	Modality	Image Size	CL Scenario	Norm. Study	CL Methods	CREA	Training Dynamics	Efficiency Analysis
Lenga et al. [36]	ChestX-ray14 [65], MIMIC-CXR [66]	Chest X-ray	1024×1024, variable	DIL	No	EWC, LwF.	No	No	No
Quarta et al. [67]	MedMNIST	Mixed	28×28	TIL, CIL	No	CWR* [68], iCaRL [69].	No	No	No
Derakhshani et al. [31]	MedMNIST	Microscope, CT	28×28	TIL, CIL, Cross-DIL	No	iCaRL	No	No	No
Wu et al. [34]	Skin8 [70], MedMNIST [35,71]	Dermoscopy, Mixed	[600, 1024], 28×28	CIL	No	LwF, EWC, iCaRL, GEM, DER++, WA [72], PODNet [73], DynaER [74], ACL [75], UCIR [76], L2P, DualPrompt, CODA-Prompt	No	No	No
Ours	NCT-CRC-HE-100K, CRC-HE-7K [2]	Histopathology	224×224	CIL	Yes	SI, Online EWC, LwF, DyTox, L2P, CODA-Prompt, DualPrompt, ER, DER, DER++.	Yes	Yes	Yes

Note: Compared with prior studies that mainly rely on low-resolution MedMNIST images, our benchmark evaluates higher-resolution patch-level colorectal histopathology images and covers a broader set of CL methods across the regularization-, architecture-, replay-, and prompt-based families. We also analyze normalization strategies, clinically relevant evaluations, training dynamics, and computational efficiency based on training time and memory cost. CWR*: rehearsal-free extension of CWR+ proposed by Lomonaco et al. [68] for small non-I.I.D. batches.

## Data Availability

The dataset analyzed in this study was obtained from the publicly available Zenodo record associated with the NCT-CRC-HE-100K and CRC-HE-7K collections: https://zenodo.org/records/1214456, accessed on 28 May 2026. Most of the continual learning methods evaluated in this study were implemented using the Mammoth framework; however, the original framework was extended in our study to support additional analyses not included in the standard Mammoth implementation, including dataset preparation, normalization, confusion matrices, ROC curves, misclassification analysis, loss and accuracy learning curves, and training time measurement. DyTox was evaluated using the official implementation released by the original authors. The additional and modified code developed for this study is publicly available at https://github.com/AILabLLL/CLMedical, accessed on 28 May 2026. No new data were created in this study.

## Supplementary Materials

The following supporting information can be downloaded at: https://www.mdpi.com/article/10.3390/diagnostics16111711/s1, Figure S1: Task-specific training objective curves for DualPrompt on the NCT-CRC-HE-100K and CRC-HE-7K datasets. The first row corresponds to NCT-CRC-HE-100K and the second row corresponds to CRC-HE-7K. The three columns show Task 1, Task 2, and Task 3, respectively; Figure S2: Epoch-level CIL accuracy curves for the DualPrompt method on the NCT-CRC-HE-100K and CRC-HE-7K datasets. Each panel corresponds to one evaluated task. Different colors indicate the training stage after which evaluation was performed, i.e., after learning Task 1, Task 2, or Task 3; Figure S3: Task-specific training objective curves for DER++ on the NCT-CRC-HE-100K and CRC- HE-7K datasets. The first row corresponds to NCT-CRC-HE-100K and the second row corresponds to CRC-HE-7K; Figure S4: Epoch-level CIL accuracy curves for the DER++ method on the NCT-CRC-HE-100K and CRC-HE-7K datasets. Each panel corresponds to one evaluated task, and the x-axis represents the training epoch. Different colors indicate the training stage after which evaluation was performed, i.e., after learning Task 1, Task 2, or Task 3; Figure S5: Loss Curves and Accuracy Curves for DualPrompt on the CRC-HE-7K Dataset; Figure S6: Loss Curves and Accuracy Curves for DER++ on the CRC-HE-7K Dataset; Table S1: Total Training Time for the Representative Continual Learning Methods.

## Abbreviations

The following abbreviations are used in this manuscript:

DualPrompt	Dual Prompting for Rehearsal-Free Continual Learning (DualPrompt)
CODA-Prompt	Continual Decomposed Attention-Based Prompting (CODA-Prompt)
CIL	Class-Incremental Learning
TIL	Task-Incremental Learning
DIL	Domain-Incremental Learning
CDIL	Cross-Domain Incremental Learning
H&E	Hematoxylin and Eosin
DER	Dark Experience Replay
DyTox	Dynamic Token Expansion
SAB	Self-Attention Blocks
TAB	Task Attention Blocks
L2P	Learning to Prompt (L2P)
SGD	Stochastic Gradient Descent
CL	Continual Learning
CF	Catastrophic Forgetting
SI	Synaptic Intelligence
ER	Experience Replay

## References

[1] Alom M.Z., Yakopcic C., Nasrin M.S., Taha T.M., Asari V.K. (2019). Breast Cancer Classification from Histopathological Images with Inception Recurrent Residual Convolutional Neural Network. J. Digit. Imaging.

[2] Kather J.N., Halama N., Marx A. 100,000 histological images of human colorectal cancer and healthy tissue. *Zenodo*
**2018**, version v0.1. https://zenodo.org/records/1214456.

[3] Causey J.L., Guan Y., Dong W., Walker K., Qualls J.A., Prior F., Huang X. (2019). Lung cancer screening with low-dose CT scans using a deep learning approach. arXiv.

[4] Rajpurkar P., Irvin J., Zhu K., Yang B., Mehta H., Duan T., Ding D., Bagul A., Langlotz C., Shpanskaya K. (2017). CheXNet: Radiologist-Level Pneumonia Detection on Chest X-rays with Deep Learning. arXiv.

[5] Ronneberger O., Fischer P., Brox T. U-Net: Convolutional Networks for Biomedical Image Segmentation. Proceedings of the MICCAI.

[6] Baumgartner C.F., Kamnitsas K., Matthew J., Fletcher T.P., Smith S., Koch L.M., Kainz B., Rueckert D. SonoNet: Real-time detection of standard scan planes in fetal ultrasound. Proceedings of the MICCAI.

[7] Taqi S.A., Sami S.A., Sami L.B., Zaki S.A. (2018). A review of artifacts in histopathology. J. Oral Maxillofac. Pathol..

[8] Shmatko A., Ghaffari Laleh N., Gerstung M., Kather J.N. (2022). Artificial intelligence in histopathology: Enhancing cancer research and clinical oncology. Nat. Cancer.

[9] Komura D., Ishikawa S. (2018). Machine learning methods for histopathological image analysis. Comput. Struct. Biotechnol. J..

[10] Kather J.N., Weis C.A., Bianconi F., Melchers S.M., Schad L.R., Gaiser T., Marx A., Zöllner F.G. (2016). Multi-class texture analysis in colorectal cancer histology. Sci. Rep..

[11] Qu H., Rahmani H., Xu L., Williams B., Liu J. (2021). Recent advances of continual learning in computer vision: An overview. arXiv.

[12] Kumari P., Chauhan J., Bozorgpour A., Huang B., Azad R., Merhof D. (2025). Continual learning in medical image analysis: A comprehensive review of recent advancements and future prospects. Med. Image Anal..

[13] Li Z., Hoiem D. (2017). Learning without forgetting. IEEE Trans. Pattern Anal. Mach. Intell..

[14] Schwarz J., Czarnecki W., Luketina J., Grabska-Barwinska A., Teh Y.W., Pascanu R., Hadsell R. Progress & compress: A scalable framework for continual learning. Proceedings of the International Conference on Machine Learning. PMLR.

[15] Rolnick D., Ahuja A., Schwarz J., Lillicrap T., Wayne G. (2019). Experience replay for continual learning. Adv. Neural Inf. Process. Syst..

[16] Buzzega P., Boschini M., Porrello A., Abati D., Calderara S. (2020). Dark Experience for General Continual Learning: A Strong, Simple Baseline. Adv. Neural Inf. Process. Syst..

[17] Douillard A., Ramé A., Couairon G., Cord M. Dytox: Transformers for continual learning with dynamic token expansion. Proceedings of the IEEE/CVF Conference on Computer Vision and Pattern Recognition.

[18] Janson P., Zhang W., Aljundi R., Elhoseiny M. (2022). A simple baseline that questions the use of pretrained-models in continual learning. arXiv.

[19] Zhang G., Wang L., Kang G., Chen L., Wei Y. Slca: Slow learner with classifier alignment for continual learning on a pre-trained model. Proceedings of the IEEE/CVF International Conference on Computer Vision.

[20] Wang Z., Zhang Z., Lee C.Y., Zhang H., Sun R., Ren X., Su G., Perot V., Dy J., Pfister T. Learning to prompt for continual learning. Proceedings of the IEEE/CVF Conference on Computer Vision and Pattern Recognition.

[21] Wang Z., Zhang Z., Ebrahimi S., Sun R., Zhang H., Lee C.Y., Ren X., Su G., Perot V., Dy J. (2022). Dualprompt: Complementary prompting for rehearsal-free continual learning. Proceedings of the European Conference on Computer Vision.

[22] Smith J.S., Karlinsky L., Gutta V., Cascante-Bonilla P., Kim D., Arbelle A., Panda R., Feris R., Kira Z. Coda-prompt: Continual decomposed attention-based prompting for rehearsal-free continual learning. Proceedings of the IEEE/CVF Conference on Computer Vision and Pattern Recognition.

[23] Kaissis G.A., Makowski M.R., Rückert D., Braren R.F. (2020). Secure, privacy-preserving and federated machine learning in medical imaging. Nat. Mach. Intell..

[24] Tabibu S., Vinod P., Jawahar C. (2019). Pan-Renal Cell Carcinoma classification and survival prediction from histopathology images using deep learning. Sci. Rep..

[25] Ulyanov D., Vedaldi A., Lempitsky V. (2016). Instance normalization: The missing ingredient for fast stylization. arXiv.

[26] Macenko M., Niethammer M., Marron J.S., Borland D., Woosley J.T., Guan X., Schmitt F., Barker J. (2009). A method for normalizing histology slides for quantitative analysis. Proceedings of the 2009 IEEE International Symposium on Biomedical Imaging: From Nano to Macro.

[27] Kirkpatrick J., Pascanu R., Rabinowitz N., Veness J., Desjardins G., Rusu A.A., Milan K., Quan J., Ramalho T., Grabska-Barwinska A. (2017). Overcoming catastrophic forgetting in neural networks. Proc. Natl. Acad. Sci. USA.

[28] Mermillod M., Bugaiska A., Bonin P. (2013). The stability-plasticity dilemma: Investigating the continuum from catastrophic forgetting to age-limited learning effects. Front. Psychol..

[29] Kim D., Han B. On the Stability-Plasticity Dilemma of Class-Incremental Learning. Proceedings of the IEEE/CVF Conference on Computer Vision and Pattern Recognition.

[30] Van de Ven G.M., Tolias A.S. (2019). Three scenarios for continual learning. arXiv.

[31] Derakhshani M.M., Najdenkoska I., van Sonsbeek T., Zhen X., Mahapatra D., Worring M., Snoek C.G. (2022). Lifelonger: A benchmark for continual disease classification. Proceedings of the International Conference on Medical Image Computing and Computer-Assisted Intervention.

[32] Ayromlou S., Tsang T., Abolmaesumi P., Li X. (2024). CCSI: Continual Class-Specific Impression for data-free class incremental learning. Med. Image Anal..

[33] Wang L., Zhang X., Su H., Zhu J. (2024). A Comprehensive Survey of Continual Learning: Theory, Method and Application. IEEE Trans. Pattern Anal. Mach. Intell..

[34] Wu X., Xu Z., Tong R.K.Y. (2024). Continual learning in medical image analysis: A survey. Comput. Biol. Med..

[35] Yang J., Shi R., Wei D., Liu Z., Zhao L., Ke B., Pfister H., Ni B. (2023). MedMNIST v2-A large-scale lightweight benchmark for 2D and 3D biomedical image classification. Sci. Data.

[36] Lenga M., Schulz H., Saalbach A. Continual learning for domain adaptation in chest X-ray classification. Proceedings of the Medical Imaging with Deep Learning. PMLR.

[37] Liu P., Yuan W., Fu J., Jiang Z., Hayashi H., Neubig G. (2023). Pre-train, prompt, and predict: A systematic survey of prompting methods in natural language processing. ACM Comput. Surv..

[38] Raghu M., Unterthiner T., Kornblith S., Zhang C., Dosovitskiy A. (2021). Do vision transformers see like convolutional neural networks?. Adv. Neural Inf. Process. Syst..

[39] Singh A., Gurbuz M.B., Gantha S.S., Jasti P. (2023). Class-incremental continual learning for general purpose healthcare models. arXiv.

[40] Kong Y., Liu L., Wang Z., Tao D. (2022). Balancing stability and plasticity through advanced null space in continual learning. Proceedings of the European Conference on Computer Vision.

[41] Zenke F., Poole B., Ganguli S. Continual learning through synaptic intelligence. Proceedings of the International conference on machine learning. PMLR.

[42] Vitter J.S. (1985). Random sampling with a reservoir. ACM Trans. Math. Softw..

[43] Dosovitskiy A. (2020). An image is worth 16x16 words: Transformers for image recognition at scale. arXiv.

[44] Touvron H., Cord M., Sablayrolles A., Synnaeve G., Jégou H. Going deeper with image transformers. Proceedings of the IEEE/CVF international Conference on Computer Vision.

[45] Hamida A.B., Devanne M., Weber J., Truntzer C., Derangère V., Ghiringhelli F., Forestier G., Wemmert C. (2021). Deep learning for colon cancer histopathological images analysis. Comput. Biol. Med..

[46] Ghosh S., Bandyopadhyay A., Sahay S., Ghosh R., Kundu I., Santosh K. (2021). Colorectal histology tumor detection using ensemble deep neural network. Eng. Appl. Artif. Intell..

[47] Sharkas M., Attallah O. (2024). Color-CADx: A deep learning approach for colorectal cancer classification through triple convolutional neural networks and discrete cosine transform. Sci. Rep..

[48] Mammoth Team Mammoth: A PyTorch Framework for Benchmarking Continual Learning. https://github.com/aimagelab/mammoth.

[49] Frascaroli E., Panariello A., Buzzega P., Bonicelli L., Porrello A., Calderara S. (2024). Clip with generative latent replay: A strong baseline for incremental learning. arXiv.

[50] Panariello A., Frascaroli E., Buzzega P., Bonicelli L., Porrello A., Calderara S. (2025). Modular embedding recomposition for incremental learning. arXiv.

[51] Boschini M., Bonicelli L., Buzzega P., Porrello A., Calderara S. (2022). Class-Incremental Continual Learning into the eXtended DER-verse. IEEE Trans. Pattern Anal. Mach. Intell..

[52] Kather J.N., Krisam J., Charoentong P., Luedde T., Herpel E., Weis C.A., Gaiser T., Marx A., Valous N.A., Ferber D. (2019). Predicting survival from colorectal cancer histology slides using deep learning: A retrospective multicenter study. PLoS Med..

[53] Wozniakova M., Skarda J., Raska M. (2022). The Role of Tumor Microenvironment and Immune Response in Colorectal Cancer Development and Prognosis. Pathol. Oncol. Res..

[54] Díaz-Rodríguez N., Lomonaco V., Filliat D., Maltoni D. (2018). Don’t Forget, There is More than Forgetting: New Metrics for Continual Learning. arXiv.

[55] Castro F.M., Marín-Jiménez M.J., Guil N., Schmid C., Alahari K. End-to-end incremental learning. Proceedings of the European Conference on Computer Vision (ECCV).

[56] Wu Y., Chen Y., Wang L., Ye Y., Liu Z., Guo Y., Fu Y. Large scale incremental learning. Proceedings of the IEEE/CVF Conference on Computer Vision and Pattern Recognition.

[57] Raghu M., Zhang C., Kleinberg J., Bengio S. (2019). Transfusion: Understanding Transfer Learning for Medical Imaging. Adv. Neural Inf. Process. Syst..

[58] Fawcett T. (2006). An introduction to ROC analysis. Pattern Recognit. Lett..

[59] Hand D.J., Till R.J. (2001). A simple generalisation of the area under the ROC curve for multiple class classification problems. Mach. Learn..

[60] Leibig C., Allken V., Ayhan M.S., Berens P., Wahl S. (2017). Leveraging uncertainty information from deep neural networks for disease detection. Sci. Rep..

[61] U.S. Department of Health and Human Services The HIPAA Privacy Rule. https://www.hhs.gov/hipaa/for-professionals/privacy/index.html.

[62] Centers for Disease Control and Prevention Health Insurance Portability and Accountability Act of 1996 (HIPAA). https://www.cdc.gov/phlp/php/resources/health-insurance-portability-and-accountability-act-of-1996-hipaa.html.

[63] U.S. Food and Drug Administration Artificial Intelligence in Software as a Medical Device. https://www.fda.gov/medical-devices/software-medical-device-samd/artificial-intelligence-software-medical-device.

[64] U.S. Food and Drug Administration Clinical Decision Support Software: Guidance for Industry and Food and Drug Administration Staff. https://www.fda.gov/regulatory-information/search-fda-guidance-documents/clinical-decision-support-software.

[65] Wang X., Peng Y., Lu L., Lu Z., Bagheri M., Summers R.M. ChestX-ray8: Hospital-scale Chest X-ray Database and Benchmarks on Weakly-Supervised Classification and Localization of Common Thorax Diseases. Proceedings of the IEEE Conference on Computer Vision and Pattern Recognition.

[66] Johnson A.E.W., Pollard T.J., Berkowitz S.J., Greenbaum N.R., Lungren M.P., Deng C.y., Mark R.G., Horng S. (2019). MIMIC-CXR, a de-identified publicly available database of chest radiographs with free-text reports. Sci. Data.

[67] Quarta A., Bruno P., Calimeri F. Continual Learning for medical image classification. Proceedings of the HC@AIxIA.

[68] Lomonaco V., Maltoni D., Pellegrini L. Rehearsal-Free Continual Learning over Small Non-I.I.D. Batches. Proceedings of the IEEE/CVF Conference on Computer Vision and Pattern Recognition Workshops.

[69] Rebuffi S.A., Kolesnikov A., Sperl G., Lampert C.H. iCaRL: Incremental Classifier and Representation Learning. Proceedings of the IEEE Conference on Computer Vision and Pattern Recognition.

[70] Tschandl P., Rosendahl C., Kittler H. (2018). The HAM10000 dataset, a large collection of multi-source dermatoscopic images of common pigmented skin lesions. Sci. Data.

[71] Yang J., Shi R., Ni B. (2021). MedMNIST Classification Decathlon: A Lightweight AutoML Benchmark for Medical Image Analysis. Proceedings of the 2021 IEEE 18th International Symposium on Biomedical Imaging (ISBI).

[72] Zhao B., Xiao X., Gan G., Zhang B., Xia S. Maintaining Discrimination and Fairness in Class Incremental Learning. Proceedings of the IEEE/CVF Conference on Computer Vision and Pattern Recognition.

[73] Douillard A., Cord M., Ollion C., Robert T., Valle E. (2020). PODNet: Pooled Outputs Distillation for Small-Tasks Incremental Learning. Proceedings of the Computer Vision–ECCV 2020.

[74] Yan S., Xie J., He X. DER: Dynamically Expandable Representation for Class Incremental Learning. Proceedings of the IEEE/CVF Conference on Computer Vision and Pattern Recognition.

[75] Zhang W., Huang Y., Zhang T., Zou Q., Zheng W.S., Wang R. Adapter Learning in Pretrained Feature Extractor for Continual Learning of Diseases. Proceedings of the Medical Image Computing and Computer Assisted Intervention–MICCAI 2023.

[76] Hou S., Pan X., Loy C.C., Wang Z., Lin D. Learning a Unified Classifier Incrementally via Rebalancing. Proceedings of the IEEE/CVF Conference on Computer Vision and Pattern Recognition.

