# Supplementary material for: Continual Learning for Histopathology Image Classification in Class-Incremental Learning
Source: Diagnostics (Basel). 2026 Jun 2;16(11):1711. doi: 10.3390/diagnostics16111711 (PMC13256828; doi:10.3390/diagnostics16111711)
Supplement: Supplementary file 1 [file diagnostics-16-01711-s001.zip › diagnostics-4254670-supplementary.pdf]

diagnostics

MDPI

Article

Continual Learning for Histopathology Image Classification in Class-Incremental Learning

Yuanyuan Wu, Yu Zhao and Anca Ralescu *

Department of Computer Science, University of Cincinnati, 2901 Woodside Drive, Cincinnati, OH 45221, USA; wu3yy@mail.uc.edu (Y.W.); zhao3y3@ucmail.uc.edu (Y.Z.)
* Correspondence: ralescal@ucmail.uc.edu

Abstract

Background: Continual learning (CL) is increasingly important for developing adaptive clinical AI models; however, its application to histopathology remains challenging due to privacy constraints, expanding diagnostic categories, and staining variability. We investigate CL for histopathology image classification under a class-incremental learning (CIL) scenario, where new diagnostic categories are introduced sequentially. **Methods**: We benchmark representative regularization-, replay-, architecture-, and prompt-based CL methods on the NCT-CRC-HE-100K dataset, with additional validation on CRC-HE-7K. We compare four normalization strategies and analyze the effects of replay buffer size and training epochs. In addition to average accuracy and forgetting, we conduct clinical relevance and error analysis using confusion matrices, ROC curves, and misclassification cases, then assess training dynamics and computational efficiency. **Results**: Dataset-level normalization consistently achieves the best performance among the evaluated normalization strategies. Among replay-based methods, DER++ achieves strong performance when previous-task images can be stored and replayed, reaching an average accuracy of 94.77 ± 1.82 and forgetting of 3.66 ± 1.73 with a buffer size of 500 and 50 training epochs. However, it requires higher memory usage, longer training time, and storage of previous samples. Among prompt-based methods, DualPrompt performs best with 5 epochs, reaching an average accuracy of 88.97 ± 0.60 and forgetting of 7.70 ± 1.21 while showing smoother training behavior and lower computational cost. **Conclusions**: Replay-based methods achieve higher accuracy and lower forgetting when exemplar storage and sufficient computational resources are available, but introduce higher computational and privacy costs. Prompt-based methods provide a competitive exemplar-free alternative under privacy- and resource-constrained settings. Dataset-level normalization is also important for stable CL performance in histopathology CIL.

Keywords: continual learning; catastrophic forgetting; class-incremental learning; prompt-based learning; digital pathology; histopathology

Check for updates

Academic Editor: Siu Wai Choi

Received: 27 March 2026
Revised: 22 May 2026
Accepted: 29 May 2026
Published: 2 June 2026

Copyright: © 2026 by the authors. Licensee MDPI, Basel, Switzerland. This article is an open access article distributed under the terms and conditions of the Creative Commons Attribution (CC BY) license.

1. Supplementary

This section presents two additional analyses to better understand the training behavior and computational cost of the two representative CL methods evaluated in this study: the prompt-based method DualPrompt and the replay-based method DER++.

1.1. Training Dynamics Analysis

To characterize the training behavior of DualPrompt and DER++, we provide two complementary analyses that operate at different granularities and serve distinct purposes. The first analysis examines **mini-batch-level training objective curves** alongside task-wise CIL accuracy curves. These are derived under the standard CL evaluation protocol and are intended to visualize optimization stability and forgetting behavior across the sequential task stream. The second analysis reports **epoch-level training–validation learning curves**, including cross-entropy loss and classification accuracy. These curves are generated using an additional held-out validation split (10% of the training data) and are used exclusively for assessing whether the models exhibit overfitting by examining the generalization gap between training and validation performance. Because the two analyses report different loss quantities, their numerical values are not directly comparable and should be interpreted independently. Specifically, the mini-batch-level curves show the training objective values used during optimization, whereas the training–validation learning curves report the standard cross-entropy loss evaluated on the task-specific training and validation splits.

1.1.1. Training Objective Curves and Forgetting Behavior

We examine optimization stability for DualPrompt on both datasets through mini-batch-level training objective curves (Figure S1), where the x-axis denotes training steps, the y-axis denotes the objective value, and vertical dashed lines indicate epoch boundaries. We report 5 epochs for DualPrompt, corresponding to the best-performing configurations identified in Section **??**. We observe that the training objective curves of DualPrompt are generally smooth on both datasets.

Across all three tasks, the training objective decreases monotonically as training progresses and eventually plateaus, indicating stable optimization behavior. On the NCT-CRC-HE-100K dataset, convergence occurs rapidly within the first few hundred steps, after which the objective stabilizes with minor oscillations. On the CRC-HE-7K dataset, the descent is more gradual and smooth throughout training, with the objective still exhibiting a slight downward trend near the end of training; however, the rate of decrease is marginal, suggesting that optimization has largely converged.

It is worth noting that some objective values are negative. This formulation is consistent with the DualPrompt loss described in the main manuscript. Specifically, the total training objective includes a prompt-matching term $\mathcal{L}_{\text{match}}(x, k_t)$, which is implemented as the negative cosine similarity between the query feature $q(x)$ and the prompt key k_t, i.e., $\mathcal{L}_{\text{match}}(x, k_t) = -\,\text{sim}(q(x), k_t)$. The total objective can therefore be expressed as:

$$\mathcal{L}_{\text{total}} = \mathcal{L}_{\text{CE}} - \lambda\ \text{sim}(q(x), k_t) \tag{1}$$

As the cross-entropy loss decreases and the query–key similarity increases over training, the total objective can take negative values. This behavior is expected and does not indicate an invalid optimization process; rather, the consistently decreasing and stabilizing trends confirm normal convergence.

The task-wise CIL accuracy curves in Figure S2 further illustrate the CL behavior of DualPrompt. A performance drop can be observed on previously learned tasks after new tasks are introduced, particularly in the Task 1 panel and, to a lesser extent, in the Task 2 panel, indicating forgetting during sequential learning. Meanwhile, Tasks 1 and 2 reach relatively stable performance within the observed training range, whereas the Task 3 curve suggests that the newly introduced task may continue to improve with additional training.

Additionally, we provide the task-specific training loss curves of DER++ on both datasets, as shown in Figure S3. Compared with the prompt-based methods, DER++ exhibits more frequent fluctuations and occasional spikes, especially on the NCT-CRC-

https://doi.org/10.3390/diagnostics16111711

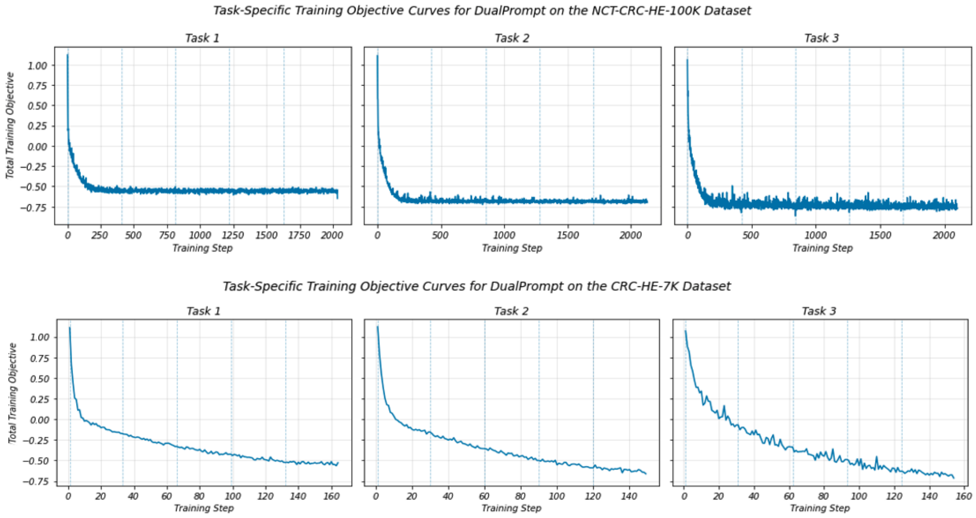

Figure S1. Task-specific training objective curves for DualPrompt on the NCT-CRC-HE-100K and CRC-HE-7K datasets. The first row corresponds to NCT-CRC-HE-100K and the second row corresponds to CRC-HE-7K. The three columns show Task 1, Task 2, and Task 3, respectively.

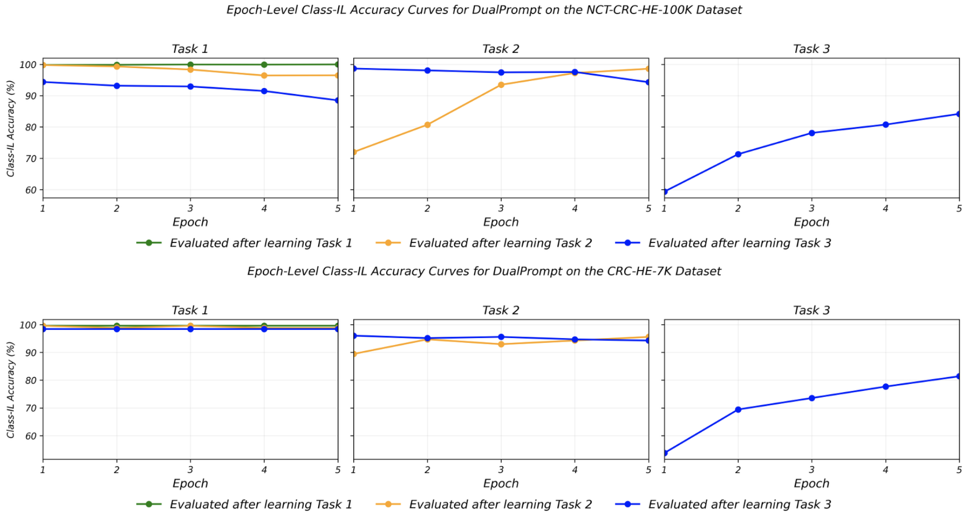

Figure S2. Epoch-level CIL accuracy curves for the DualPrompt method on the NCT-CRC-HE-100K and CRC-HE-7K datasets. Each panel corresponds to one evaluated task. Different colors indicate the training stage after which evaluation was performed, i.e., after learning Task 1, Task 2, or Task 3.

https://doi.org/10.3390/diagnostics16111711

HE-100K dataset. On CRC-HE-7K, the loss curve for Task 1 is relatively smooth, while Tasks 2 and 3 show occasional spikes. In contrast, on NCT-CRC-HE-100K, spikes appear more frequently across all three tasks, particularly in Tasks 1 and 3. This behavior does not necessarily indicate invalid or failed training; rather, it reflects the stochastic nature of replay-based optimization and the multi-term DER++ objective.

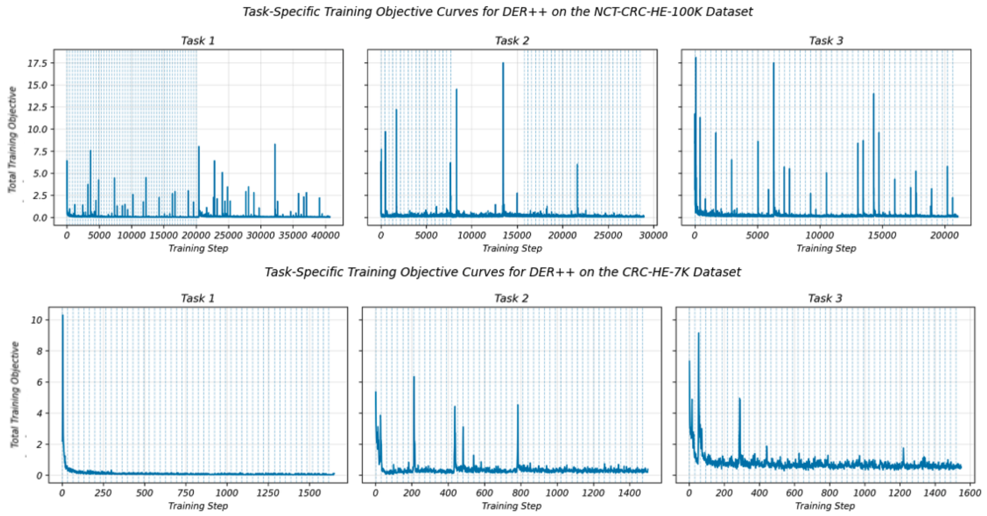

Figure S3. Task-specific training objective curves for DER++ on the NCT-CRC-HE-100K and CRC-HE-7K datasets. The first row corresponds to NCT-CRC-HE-100K and the second row corresponds to CRC-HE-7K.

Specifically, DER++ optimizes a composite objective that combines the classification loss on the current mini-batch, replay classification loss on buffered samples, and logit-matching loss that encourages the current model outputs on replayed samples to remain close to stored historical logits [1]. Because replay samples are randomly drawn from the memory buffer at each training step, the difficulty, class composition, and conflict between current-task samples and replayed old-task samples can vary substantially across mini-batches. As a result, the total objective may show high-frequency oscillations or sharp spikes, especially around task transitions when the data distribution changes.

The task-wise CIL accuracy curves in Figure S4 further show the sequential learning behavior of DER++. On NCT-CRC-HE-100K, larger accuracy fluctuations are observed, especially in Tasks 1 and 3, whereas this phenomenon is less pronounced on CRC-HE-7K. This suggests that DER++ optimization is more variable on the larger and more complex NCT-CRC-HE-100K dataset, where longer training trajectories and more diverse replay-buffer samples may introduce stronger mini-batch-level fluctuations. Overall, DER++ shows more fluctuating training dynamics than DualPrompt in our experiments, although it achieves strong final performance.

Overall, the training objective and accuracy curves show that DualPrompt exhibits smoother training dynamics than DER++ in our histopathology CIL experiments, whereas DER++ presents more frequent loss spikes and accuracy fluctuations due to its replay-based multi-term objective and stochastic buffer sampling.

1.1.2. Overfitting Analysis

Due to computational constraints, the training–validation learning curve analysis is conducted on the CRC-HE-7K dataset only. We compare cross-entropy loss and classification accuracy on the training and held-out validation splits across tasks to assess whether either method exhibits signs of overfitting.

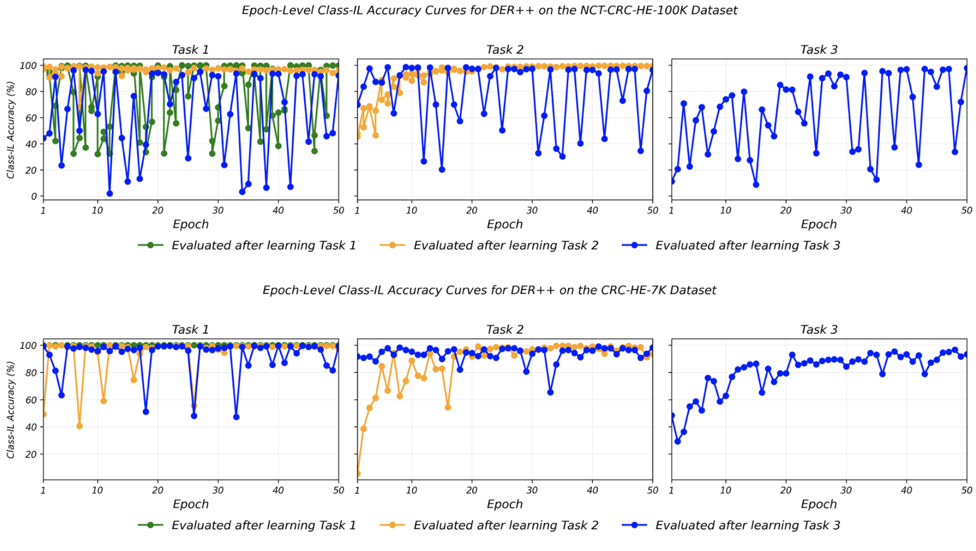

Figure S4. Epoch-level CIL accuracy curves for the DER++ method on the NCT-CRC-HE-100K and CRC-HE-7K datasets. Each panel corresponds to one evaluated task, and the x-axis represents the training epoch. Different colors indicate the training stage after which evaluation was performed, i.e., after learning Task 1, Task 2, or Task 3.

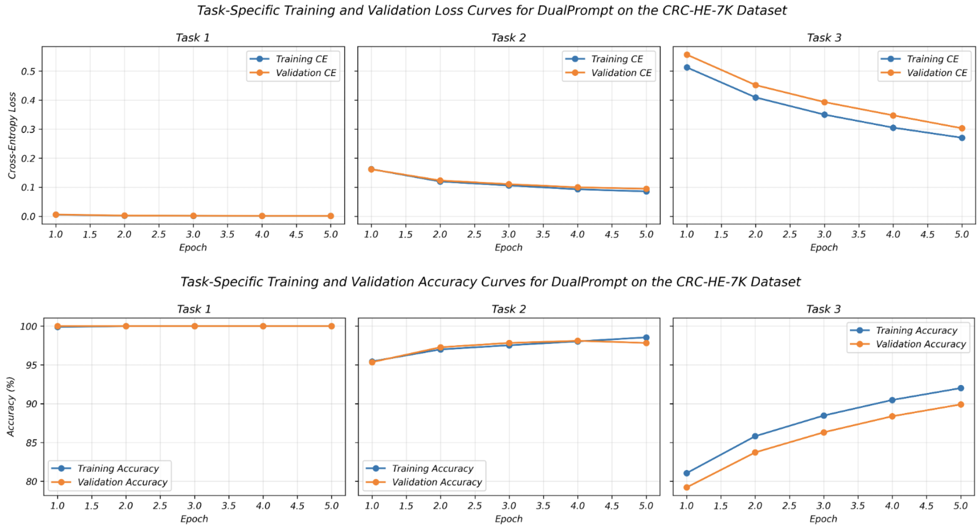

Figure S5. Loss Curves and Accuracy Curves for DualPrompt on the CRC-HE-7K Dataset

For the learning-curve analysis, we report the standard cross-entropy loss evaluated on the task-specific training and validation splits. The loss is averaged over all samples within each split. Under the CIL, the CE loss is calculated over all classes observed up to the current task, consistent with our evaluation protocol. This provides a standardized and directly comparable measure of predictive performance across the training and validation splits. We separately refer to the mini-batch values recorded during optimization as the training objective, since those values may include method-specific auxiliary terms and are therefore not used for assessing the training–validation generalization gap.

As shown in Figure S5, DualPrompt exhibits no clear signs of overfitting on Tasks 1 and 2, where training and validation loss are closely aligned and the corresponding accuracy curves nearly overlap. For Task 1, the accuracy curves are nearly saturated from the first epoch: the training accuracy increases slightly from 99.89% to 100.00%, while the validation accuracy remains at 100.00%. This very small change is difficult to distinguish at the original plotting scale. Nevertheless, the training CE decreases from 0.0053 to 0.0009 and the validation CE decreases from 0.0060 to 0.0011, indicating that optimization still occurs despite the saturated accuracy. This early saturation is likely due to the relatively small size of CRC-HE-7K and the high separability of the Task 1 tissue categories. For Task 3, a modest training–validation gap is observed in both loss and accuracy, with training accuracy increasing faster than validation accuracy. However, validation loss continues to decrease and validation accuracy continues to improve across all 5 epochs, indicating that the model is still generalizing rather than memorizing the training data. Therefore, no clear evidence of overfitting is observed for DualPrompt.

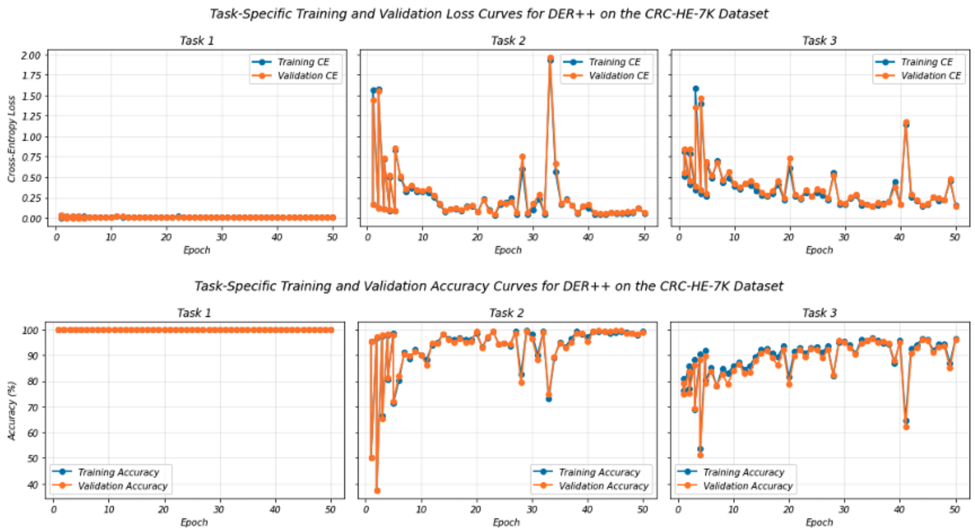

Figure S6. Loss Curves and Accuracy Curves for DER++ on the CRC-HE-7K Dataset

As shown in Figure S6, DER++ reaches near saturation on Task 1, with both training and validation CE close to zero and accuracy close to 100%. For Tasks 2 and 3, the CE curves exhibit several epoch-level spikes; however, training and validation curves fluctuate together rather than diverging systematically. This co-fluctuation pattern does not match the typical signature of overfitting, where training loss decreases while validation loss increases. The absence of a persistent training–validation divergence indicates no clear evidence of systematic overfitting for DER++.

Overall, neither DualPrompt nor DER++ exhibits clear signs of overfitting on the CRC-HE-7K dataset under the evaluated configurations.

https://doi.org/10.3390/diagnostics16111711

1.2. Training Time Analysis

The total training time was calculated by adding the final elapsed training time across all tasks recorded in the training logs. As shown in Table S1, DualPrompt required less training time than DER++ on both the NCT-CRC-HE-100K and CRC-HE-7K datasets under the experimental settings used in this study. Specifically, DualPrompt required 00:55:09 on NCT-CRC-HE-100K and 00:04:59 on CRC-HE-7K, whereas DER++ required 17:43:53 and 01:28:20, respectively.

Table S1. Total Training Time for the Representative Continual Learning Methods

Dataset	Method	Epochs	Buffer Size	Training Time
NCT-CRC-HE-100K	DualPrompt	5	-	00:55:09
	DER++	50	500	17:43:53
CRC-HE-7K	DualPrompt	5	-	00:04:59
	DER++	50	500	01:28:20

1. Buzzega, P.; Boschini, M.; Porrello, A.; Abati, D.; Calderara, S. Dark Experience for General Continual Learning: A Strong, Simple Baseline. *Adv. Neural Inf. Process. Syst.* **2020**, *33*, 15920–15930.
